# Climatic niche shifts and ecological sky‐island dynamics in Mesoamerican montane birds

**DOI:** 10.1002/ece3.70236

**Published:** 2024-09-04

**Authors:** Alberto Rocha‐Méndez, David A. Prieto‐Torres, Luis A. Sánchez‐González, Adolfo G. Navarro‐Sigüenza

**Affiliations:** ^1^ Museo de Zoología, Facultad de Ciencias Universidad Nacional Autónoma de México Mexico City Mexico; ^2^ Posgrado en Ciencias Biológicas Universidad Nacional Autónoma de México Mexico City Mexico; ^3^ Laboratorio de Biodiversidad y Cambio Global (LABIOCG), Facultad de Estudios Superiores Iztacala Universidad Nacional Autónoma de México Tlalnepantla Estado de México Mexico; ^4^ Unidad Multidisciplinaria de Docencia e Investigación, Facultad de Ciencias, Campus Juriquilla Universidad Nacional Autónoma de México Querétaro Mexico; ^5^ Present address: Evolutionary Adaptive Genomics, Institute for Biochemistry and Biology, Faculty of Mathematics and Natural Sciences University of Potsdam Potsdam Germany

**Keywords:** biogeography, ecological niche modeling, moist forest model, neotropical birds, niche conservatism

## Abstract

An ongoing challenge in evolutionary and ecological research focuses on testing biogeographic hypotheses for the understanding of both species' distributional patterns and of the factors influencing range limits. In this study, we described the climatic niches of Neotropical humid montane forest birds through the analysis of factors driving their evolution at inter‐ and intraspecific levels; and tested for differences among allopatric lineages within *Aulacorhynchus, Chlorospingus, Cardellina,* and *Eupherusa*. We employed ecological niche models (ENMs) along with an ordination approach with kernel smoothing to perform niche overlap analyses and test hypotheses of niche equivalence/similarity among lineages. In addition, we described the potential distributions of each lineage during the Late Pleistocene climate fluctuations, identifying historical range expansions, connectivity, and stability. Overall, we observed differences in environmental variables influencing climatic requirements and distributional patterns for our selected species. We detected the highest values of niche overlap mainly between *Eupherusa* and some *Chlorospingus* lineages. At both interspecific and intraspecific levels, sister lineages showed non‐identical environmental niches. Our results offer weak support to a moist forest model, in which populations followed the expansion and contraction cycles of montane forests, leading to a lack of niche conservatism among lineages (they tend to occupy not identical climatic environments) throughout Mesoamerica. Therefore, historical climatic conditions may act as ecological barriers determining the distributional ranges of these species.

## Graphical abstract

We describe climatic niche of four Neotropical montane birds through the analysis of climatic factors driving their evolution at inter‐ and intraspecific levels. We tested for differences among lineages within four taxa (*Aulacorhynchus*, *Chlorospingus*, *Cardellina*, and *Eupherusa*), all associated to Mesoamerican humid montane forests. Our results, as a combination of environmental and geographical frameworks, support that these taxa which inhabit similar areas examined at distinct hierarchy levels tend to occupy not identical, but somewhat similar, climatic environments, and the lack of niche conservatism.

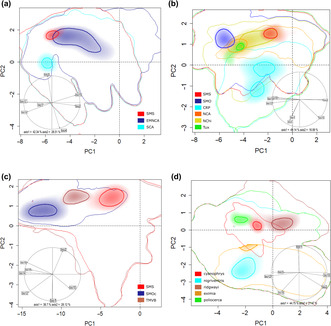

## 1 INTRODUCTION

An ongoing challenge in evolutionary and ecological research focuses on testing biogeographic hypotheses with the aim of understanding and quantifying species distributions and determining which factors influence species range limits (Carnaval et al., 2009; Schluter, 2009; Wiens & Graham, 2005). Climatic conditions may act as ecological barriers determining distributional ranges. Nevertheless, climate alone cannot always predict the distribution of a species, as many of them do not occupy all available habitats and biotic interactions such as mutualism, competition or predation can enhance or limit population processes (Broennimann et al., 2012; Elith & Leathwick, 2009; Soberón & Nakamura, 2009; Soberón & Peterson, 2005). The geographic ranges of species can be depicted as the intersection of suitable biotic and abiotic conditions that have been available and geographically accessible to its populations through time. Such conditions may be considered as one of the main drivers of evolutionary change because of their influence at different spatio‐temporal scales (Hutchinson, 1957, 1978; Rödder & Engler, 2011; Soberón, 2007; Srivastava et al., 2020). Biological interpretations of distributional patterns aim to describe the most important environmental predictors that produce and shape the areas of distribution of species. Thus, ecological niche models (ENM) and species distribution modeling (SDM) are useful to test differentiation scenarios by measuring niche overlap and evaluating niche evolution among species (McCormack et al., 2010; Peterson et al., 2011; Warren et al., 2008).

Evolutionary dynamics of the avifauna in montane regions have been the focus of research worldwide (e.g., Antonelli et al., 2018; Rahbeck et al., 2019), especially due to fragmented distributional patterns in the so called “sky‐islands”. This pattern corresponds to high‐elevation habitats geographically subdivided and isolated among different mountain ranges (McCormack et al., 2008). Montane regions are considered as biodiversity hotspots (Bertelli et al., 2017; Mastretta‐Yanes et al., 2015; Ramírez‐Barahona & Eguiarte, 2013), and available studies suggest that Quaternary climate oscillations have promoted habitat altitudinal shifts along mountain slopes (e.g., Caballero et al., 2010; Lachniet & Vázquez‐Selem, 2005). This has led to the development of the moist forest model (Ramírez‐Barahona & Eguiarte, 2013), in which highland biotas have been united through forest type corridors among previously isolated mountains, while during postglacial periods these biotas become retracted to high elevations. Under this scenario and given restricted dispersal, a pattern of isolation by distance (Slatkin, 1993) may favor the accumulation of local genetic differences. Furthermore, the phylogenetic relatedness of many taxa occurring in distinct mountains is closely related to inherent characteristics in each mountain range, such as geological history, geographic location, degree of isolation, and disturbance (Arriaga‐Jiménez et al., 2020; Mastretta‐Yanes et al., 2015; Ornelas et al., 2016; Venkatraman et al., 2018).

Several studies involving Mesoamerican montane avian taxa with isolated populations have focused on divergence times and phylogeographic breaks (e.g., Moreno‐Contreras et al., 2020; Navarro‐Sigüenza et al., 2008; Ornelas et al., 2010, 2013; Rodríguez‐Gómez & Ornelas, 2018; Vázquez‐López et al., 2020; Venkatraman et al., 2018). Given that differentiation resulting from habitat isolation‐connection cycles is often coupled with the ecological divergence of populations, we aim to explore the potential changes in distributional patterns and ecological niche overlap among lineages of bird taxa inhabiting humid montane forests in Mesoamerica (e.g., Alcántara et al., 2002; Moreno‐Contreras et al., 2020; Ornelas et al., 2013). These lineages are represented by largely allopatric and differentiated populations restricted to the main mountain ranges in the area (sky islands sensu McCormack et al., 2008), whose dispersal abilities are limited. Isolation may also be associated with differences in climatic regimes (Moreno‐Contreras et al., 2020). Therefore, these taxa represent a suitable model to analyze the role that niche conservatism and environmental overlap may have had in population divergence and assess whether niche divergence accompanies evolutionary differentiation as suggested by previous studies with other bird taxa (Castillo‐Chora et al., 2021a; Moreno‐Contreras et al., 2020; Vázquez‐López et al., 2020).

Here, we studied niche evolution in four unrelated Mesoamerican avian taxa and assessed whether their distributional patterns and evolutionary divergence are influenced by common environmental factors. We specifically aimed to (1) characterize differences/similitudes of the Grinnellian niche (Rödder & Engler, 2011) among lineages; (2) test whether intraspecific lineages are more ecologically similar throughout their distribution as the ecological requirements for their successful establishment and persistence can be characterized using the concept of overlap; (3) to examine whether congruence between the ecological and geographical variations among species corresponds to the presence of past barriers to gene flow by estimating palaeodistributions during the Quaternary; and (4) test whether patterns of niche evolution of intraspecific lineages are consistent among taxa. We assume that isolated lineages will exhibit patterns of niche segregation (null or low climatic niche overlap), as a result of differentiated climatic regimes acting on their distributional ranges, whereas lineages inhabiting the same region will show larger climatic niche overlap, as consequence of similar environmental pressures acting through their evolutionary history.

## 2 MATERIALS AND METHODS

### 2.1 Data gathering and comparison units

We selected taxa in the avian genera *Aulacorhynchus* (Ramphastidae, *n = 4*), *Chlorospingus* (Passerellidae, *n = 6*), *Cardellina* (Parulidae, *n = 3*), and *Eupherusa* (Trochilidae, *n = 4*). All of them are largely sedentary and restricted to isolated patches of humid montane forest in Mesoamerica (Hernández Baños et al., 1995), they also represent diverse ecological attributes (frugivores, insectivores, and nectarivores), and which genetic and phylogeographic discontinuities are well documented (Avendaño et al., 2013; Barrera‐Guzmán et al., 2012; Bonaccorso et al., 2008, 2011; Bonaccorso & Guayasamin, 2013; García‐Moreno et al., 2004; Hernández‐Baños et al., 2012; Maldonado‐Sánchez et al., 2016; Navarro‐Sigüenza et al., 2001; Puebla‐Olivares et al., 2008; Sánchez‐González et al., 2007; Weir et al., 2008; Winker, 2016).

We compiled species occurrence records from the Global Biodiversity Information Facility database (GBIF; http://www.gbif.org; Appendix S1, Table S2) and the “Atlas of distribution of Mexican birds” (Navarro‐Sigüenza et al., 2003; Peterson et al., 2016). Due to shortcomings in the GBIF data (Nori et al., 2023; Yesson et al., 2007) and the need of good quality data for model performance, we checked the database to remove erroneous, geographically doubtful, and duplicate records. Also, because most records are collected opportunistically, and some areas are disproportionally surveyed leading to spatially auto‐correlated clusters of records (Peterson et al., 2015), for each species we applied a buffer distance of 10 km^2^ to avoid sampling biases and retained only these records corresponding to localities separated by this buffer distance using the “NTBOX” R library (Osorio‐Olvera et al., 2020). Our clean dataset contained 592 unique occurrence records for our selected species as follows: *Aulacorhynchus prasinus* (Emerald Toucanet, *n* = 157 records), *Chlorospingus flavopectus* (Common Chlorospingus, *n* = 200), *Cardellina rubra* (Red warbler, *n* = 108), *Eupherusa cyanophrys* (Blue‐capped Hummingbird, *n* = 7), *E. eximia* (Stripe‐tailed Hummingbird, *n* = 51), *E. nigriventris* (Black‐bellied Hummingbird, *n* = 48), *E. poliocerca* (White‐tailed Hummingbird, *n* = 10), and *E. ridgwayi* (Mexican Woodnymph, *n* = 11). All geographic coordinates were transformed to decimal degrees, based on the WGS84 datum.

**FIGURE 1:**
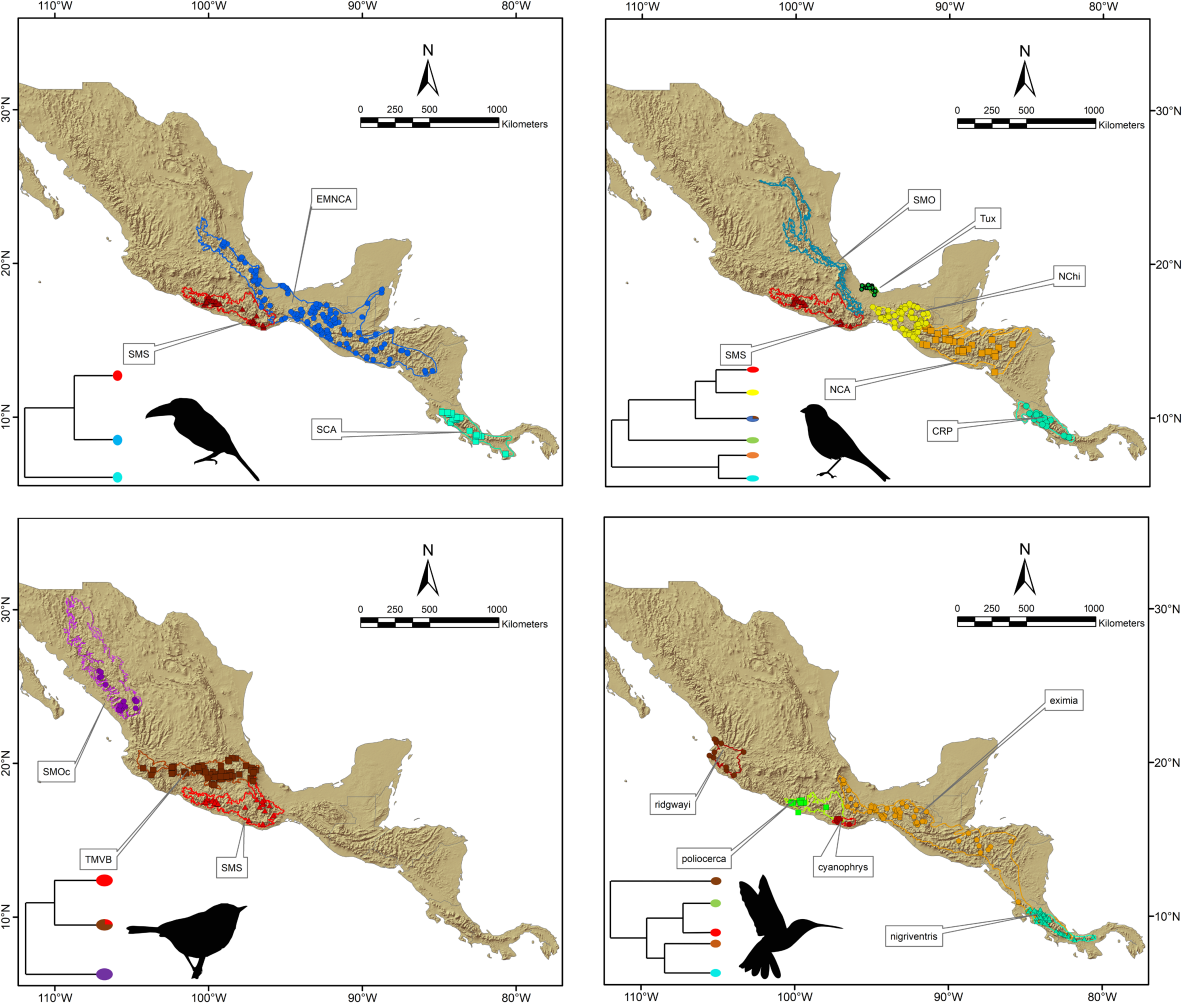
Summary of occurrence data, distributional patterns, and phylogenetic relationships of our selected lineages (according to Rocha‐Méndez et al., 2019). Colored circles at the end of the branches in the phylogenetic trees correspond to the occurrence records for each lineage. Maps represent the accessibility areas (“*M*”) for each lineage. Bird silhouettes represent the taxon in each map: *Aulacorhynchus* (top left), *Chlorospingus* (top right), *Cardellina* (bottom left), *Eupherusa* (bottom right).

Due to the implicit and indissoluble relationship between niche and species in ENM approaches (Soberón & Nakamura, 2009), the criteria for grouping occurrence data into a “species” category becomes very important to accurately define the question of interest (Funk et al., 2012). Herein, units selected to establish intraspecific comparisons within each taxon were defined based on lineages previously recognized in genetic analyses, each of which appears to be restricted to a single mountain range in Mesoamerica (e.g., Barrera‐Guzmán et al., 2012; Bonaccorso et al., 2008, 2011; Hernández‐Baños et al., 2012; Rocha‐Méndez et al., 2019; Weir et al., 2008). Assignment of occurrence data to lineages (Figure 1) resulted in *A. prasinus* (eastern Mexico and northern Central America‐EMNCA; *n* = 124), *A*. *prasinus* (southern Central America‐SCA; *n* = 19), *A. prasinus* (Sierra Madre del Sur‐SMS; *n* = 14); *C. flavopectus* (Costa Rica Panama‐CRP; *n* = 22), *C. flavopectus* (northern Central America‐NCA; *n* = 34), *C. flavopectus* (Sierra Madre Oriental‐SMO; *n* = 52), *C. flavopectus* (Tuxtlas massif‐Tux; *n* = 12), *C. flavopectus* (northern Chiapas‐NChi; *n* = 63), *C. flavopectus* (Sierra Madre del Sur‐SMS; *n* = 17), *C. rubra* (TMVB; *n* = 68), *C. rubra* (Sierra Madre Occidental‐SMOc; *n* = 18), and *C. rubra* (Sierra Madre del Sur‐SMS; *n* = 22). *Eupherusa* occurrence records were not subdivided into lineages.

### 2.2 Defining accessibility areas

We defined an accessible area for each lineage (“*M*” sensu Soberón & Peterson, 2005), to establish the area where ecological niches are projected on both the environmental (E‐space) and geographic spaces (G‐space). These areas and their extent correspond to accessible areas for the species in each time span and must be customized for each involved taxon based on biogeographic and ecological features (Barve et al., 2011). Herein, we delimited the accessible area for each lineage as those grid cells within the terrestrial ecoregions (Dinerstein et al., 2017) across Mesoamerica where historical occurrence records are found. To do this, we intercepted the occurrence records and the terrestrial ecoregions, to which we added a buffer of 30 km around each point and obtained a polygon in ArcMap 10.8.2 (ESRI, 2010). This buffer value was obtained as the mean distance among the occurrence records of each lineage analyzed herein. Such consideration assumed that the defined regions represent the lineages environmental tolerance limits and ecological barriers to dispersal in geographical space and thus could act as potentially invasive areas for geographic projection in different times.

### 2.3 Current and paleoclimate data

We obtained environmental variables from interpolated climate data available in WorldClim v. 1.4 (Fick & Hijmans, 2017) and CHELSA v.1.0 (Karger et al., 2017). These bioclimate data encompass a ≥ 30‐year period for 19 variables summarizing aspects of precipitation and temperature with 30″ of resolution (~1 km^2^ cell size). Herein, we decided to use both datasets for testing the consistency of overlap niche results (see below) and, therefore, reduce the uncertainties in forecasting into models. Nevertheless, because we did not observe differences in the main trends identified for the niche overlap comparisons among lineages, we only show the WorldClim results (analyses performed with the CHELSA dataset are in: https://github.com/rochamendez/ClimaticNicheShifts_MesoamericanBirds).

To avoid over‐fitting in the models (see Peterson et al., 2011) and reduce the multicollinearity problem (Broennimann et al., 2012; Regos et al., 2019; Srivastava et al., 2020), we reduced the number of variables used in the analyses using a cluster dendrogram based on Pearson correlations, as implemented in the “NTBOX” library (Osorio‐Olvera et al., 2020) for R. However, to conduct a multi‐species niche analysis in the E‐space, we first sought to discover if all species shared the same set of climatic variables that could best explain their ecological requirements. We observed that each species responds to unique ecologically and physiologically relevant variables (Appendix S1, Figures S1–S3; Appendix S2, Tables S1–S17), preventing the choice of a single set of variables to include in multi‐species niche analyses (e.g., Moreno‐Contreras et al., 2020; Sánchez‐González et al., 2023). Therefore, we defined different subsets of bioclimatic variables for representation of niches for each lineage and performed the correlation analyses on them to retain their correspondent uncorrelated bioclimatic variables. Despite correlation coefficients of 0.28 have been reported to cause model overfitting (Graham, 2003), we used a conservative Pearson correlation coefficient of *r* ≤ .8. Then, we proceeded to perform niche overlap and equivalency analyses. Individual species uncorrelated variables were further used for distribution model projections.

For distributional models based on past climate projections, we used Late Pleistocene climate data scenarios (Last Interglacial [LIG; 120,000–140,000 ya], Last Glacial Maximum [LGM; ca. 22,000 ya], and the Mid‐Holocene [ca. 6000 ya]; Otto‐Bliesner et al., 2006). We used layers from two global climate circulation models: the Community Climate System Model (CCSM4; Gent, 2011) and the Interdisciplinary Research on Climate Earth System Model (MIROC‐ESM; Watanabe et al., 2011). All climatic variables and scenarios were stored in an ASCII “raster” format and imported into ArcMap 10.8.2 (ESRI, 2010) with a spatial resolution of 1km^2^.

### 2.4 Niche overlap in E‐space and statistical niche dynamics

We analyzed the role of ecological conditions in the divergence of bird lineages estimating the niche equivalence/similarity among them using a principal component analysis (PCA‐env) and *Schoener's D* (Schoener, 1970; Warren et al., 2008). To do this, we performed the ordination methods developed by Broennimann et al. (2012), as implemented in the “ecospat” R library (Di Cola et al., 2017). This method corrects for potential bias sampling by considering the available environmental space (E‐space) within the entire background applying a kernel smoothing function to occurrence densities (Broennimann et al., 2012; Silva et al., 2016). Results from niche overlap (ranging from 0 [complete dissimilarity] to 1 [complete overlapping]) are shown based on the proposal by Rödder and Engler (2011): null or very low overlap (0–0.2), low overlap (0.2–0.4), moderate overlap (0.4–0.6), high overlap (0.6–0.8), very high overlap (0.8–1.0).

We performed tests of niche equivalency (niches effectively indistinguishable from one another; Graham et al., 2004; Warren et al., 2008) and similarity (niche conservatism between lineages) following the proposal by Broennimann et al. (2012): (1) calculating the density of occurrences and of environmental factors (based on the uncorrelated environmental variables described previously for each species‐pair) along the environmental axes of PCA‐env; (2) estimating the niche overlap along the gradient of multivariate analysis; and (3) performing statistical tests to compare the empirically observed distributions of *Schoener*'s *D* to 100 randomly generated simulated values (see Broennimann et al., 2012; Warren et al., 2008 for a complete explanation). From this latter step, a histogram of the null distribution is constructed (Appendix S1, Figures S4 and S5). Then, the hypotheses of niche equivalence and/or similarity are accepted when the empirically observed *D* values are significantly (*p* < .05) higher than the simulated values. The R code used for visualization of niche overlap and performing the niche equivalence/similarity tests was obtained from Broennimann et al. (2012) and Silva et al. (2016); however, we modified the original scripts to increase the number of lineages for our study. The scripts and input information used for all these analyses can be found at https://github.com/rochamendez/ClimaticNicheShifts_MesoamericanBirds.

### 2.5 Geographic analysis: Historical distribution and areas of long‐term climatic stability

We used an ENM approach (Peterson, 2011) to reconstruct the Quaternary distribution of each lineage. This approach has been successfully used to describe the potential distribution range and niche shift of a variety of species at large geographical scales (e.g., Arriaga‐Jiménez et al., 2020; Cox et al., 2019; Luna‐Aranguré et al., 2019), allowing us to identify potential areas of historical range extension, connectivity, and stability during the Late Pleistocene climate fluctuations. We used MaxEnt v.3.4.1 (Phillips et al., 2006), to obtain the potential distributions per lineage. This software calculates the probability of environmental suitability for each pixel given a sample of the background in function of occurrence localities and environmental variables, under the assumption that the expected value must be equal to the empirical average value of presence points (Phillips et al., 2006; Phillips & Dudík, 2008). We used MaxEnt given its good performance using presence‐only data (Elith et al., 2011), and because it allows a calibration protocol to assess model complexity by selecting the best modeling parameters (see Cobos et al., 2019; Muscarella et al., 2014).

All models were run allowing “unconstrained extrapolation” and “extrapolation by clamping” options, which allowed us to identify potential novel conditions that could be considered suitable for each species in past scenarios (Elith et al., 2011; Merow et al., 2014). Models for lineages with <15 records were developed using all presence data (using MaxEnt default parameters) and evaluated with a Jackknife test (Pearson et al., 2007). For species with ≥15 records, models were generated using the “*kuenm*” R package (Cobos et al., 2019) to perform a calibration protocol assessing model complexity (Appendix S2, Figures S1 and S2); Merow et al., 2014). For the latter, models were generated using randomly 50% of the records as training data and the remaining 50% for model evaluation (testing data). Models were first calibrated by creating 210 candidate models (per lineage), with parameterizations resulted from seven regularization multipliers (*β*: .1, .25, .5, 1, 2, 4, 6) and all the potential combinations of five feature classes (Linear, Quadratic, Product, Threshold, and Hinge responses). Best models were chosen based on omission errors (Anderson et al., 2003), the partial ROC test (Peterson et al., 2008), and the Akaike Information Criterion (AIC; Merow et al., 2014; Muscarella et al., 2014). After model calibration, we created models with the selected parameter values, 500 iterations with 10 bootstrap replicates, and *cloglog* output (Phillips et al., 2017), and transferred them to present and past environmental scenarios (see above). We calculated median values across replicates to summarize model predictions (Campbell et al., 2015) and created presence‐absence maps using as the threshold value the “tenth percentile training presence” (Liu et al., 2013), to generate the distribution maps for each species under each climate scenario. For each species, past geographic distributions were obtained manually by overlaying the binary projections from the two global climate circulation models, allotting “presence” to a pixel where both predictive models coincided.

For each taxon, we assessed the degree of geographical matching between the potential ranges for the different lineages predicted across each climatic scenario (alloprediction values), which allowed us to understand the role of geographical drivers of niche diversification among lineages across Mesoamerica. Models are expected to predict similar potential distribution ranges if the climatic niche for lineages is similarly distributed and historically congruent between them, despite geographical barriers (e.g., Mota‐Vargas & Rojas‐Soto, 2016). Finally, for each lineage we obtained long‐term climatic stability areas or refugia (pixels where a population per lineage was predicted to occur in all four climatic scenarios) by overlapping the binary maps obtained for each climatic scenario (see Terribile et al., 2012). This latter allowed us the identification of climatic stability/instability areas for the evolution and maintenance of lineage diversity (e.g., Cabanne et al., 2016; Carnaval et al., 2009; Castillo‐Chora, Sánchez‐González, et al., 2021).

## 3 RESULTS

### 3.1 Calculating niche overlap

**FIGURE 2:**
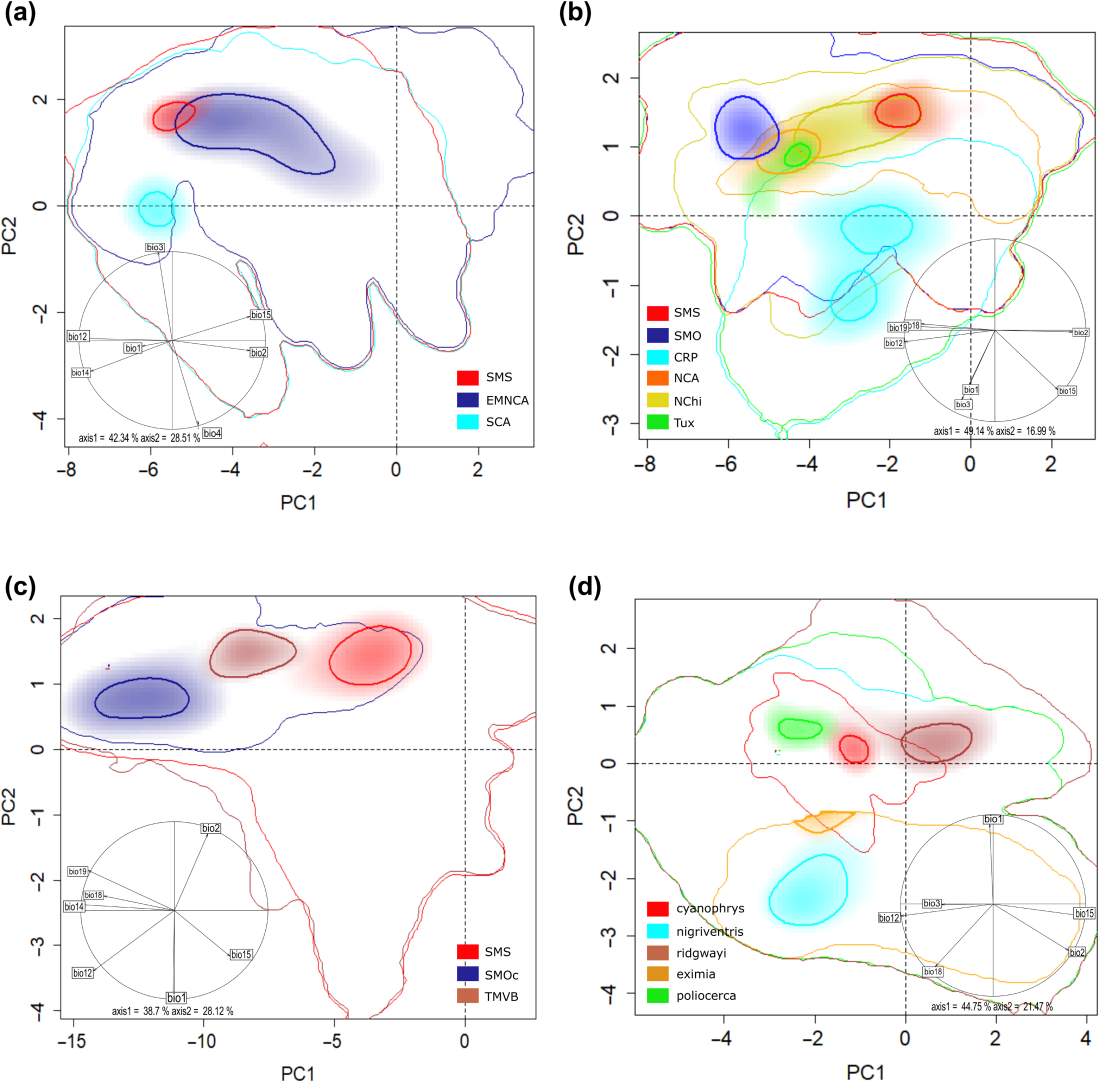
Overlap of the realized climatic niches for *Aulacorhynchus* (a), *Chlorospingus* (b), *Cardellina rubra* (c), and *Eupherusa* (d) lineages. Color shading depicts the density of occurrences for each lineage by cell. The strong contours represent 10% of the highest values of occurrence density, whereas the thin lines represent 100% of the background available in the region. The correlation circles at the lower position of the graphs show the contribution of the climatic variables on the two axes of the PCA.

PCA‐Env based on occurrence records for lineages in *Aulacorhynchus* showed that ecological niches exhibit high shift and relative expansion among populations inhabiting distinct mountain ranges (Appendix S1, Figure S6). We observed that the first two principal components explained ~ 70.8% of the total variance observed among groups (PC1 = 42.34% and PC2 = 28.51%). Overall, the smallest niche in E‐space corresponds to the SMS lineage, whereas the largest to the eastern Mexican and northern Central America lineage, which are slightly overlapped. The SCA lineage is completely separated from the latter two along PC2 (Figure 2a). According to the observed niche overlap values among these three lineages throughout their distribution, we found that the three paired comparisons had null or very limited (< 0.20 scores) overlap for *D* values. In fact, the ordination null tests of niche equivalency showed that niches overlapping values were not significantly (*p >* .05) higher than the simulated values in 100% of the paired comparisons, that is, Grinnellian niches are not identical. Likewise, the background similarity tests indicated non‐significant results for the comparison of simulated null distributions and empirical values for the *D* metric among lineages (Table 2). Thus, the hypothesis of niche similarity (conservatism) in *Aulacorhynchus* lineages can also be rejected.

We observed that for *Chlorospingus* lineages the first two principal components represented ~66% of the total variance observed (PC1 = 49.14% and PC2 = 16.99%) among groups (Figure 2b). Overall, each lineage differed in their position in the multidimensional E‐space with respect to density of their respective occurrences, thus showing marked differences in niche breadth across Mesoamerica. Nevertheless, we observed only two of the paired comparisons (CRP vs. NCA and CRP vs. SMO) with significant higher overlap *D* values than those randomly generated simulated values, this implies that in 13% of cases niche equivalence cannot be rejected because of shared environmental conditions (Table 2). Furthermore, the population from Los Tuxtlas showed the narrowest niches and appears to be nested within the NCA niche (Figure 2b; Appendix S1, Figure S7). Similarity tests within *Chlorospingus* revealed that observed niche overlapping values were not higher than the simulated values for all paired comparisons. Therefore, based on their ecological niche characteristics and occurrence data, we also rejected niche conservatism hypothesis (equivalence or similarity) in *Chlorospingus* lineages (Table 2, Figure 2b; see Appendix S1, Figures S4 and S7).

The PCA‐env analysis for *Cardellina* occurrence records (Figure 2c) exhibited high shift among niches of lineages inhabiting distinct mountain ranges. In this case, the first two principal components (PC1 = 38.7% and PC2 = 28.12%) account for 66.8% of the total variance among groups. In general, these lineages exhibited an overlapping density occurrence across PC2 but their differentiation across E‐space is due to PC1; they show very similar sizes across the E‐space (see Appendix S1, Figure S8). Niche overlap analyses also revealed an overall pattern of low and not significant *D* values among lineages (Table 2). Based on the obtained values for both niche equivalence/similarity tests, Grinnellian niches of the three lineages are not equivalent; and the niche conservatism hypothesis can also be rejected.

Finally, our PCA analysis for *Eupherusa* species showed that PC1 (44.75%) and PC2 (21.47%) represent 66.2% of the total variance (Figure 2d; Appendix S1, Figure S9). All comparisons of niche overlap indicated non‐significant and null to low values of *D* scores (Table 2). Overall, these observed overlapping values mostly fall in the tails of the null distributions for the niche equivalence test, so niches are not indistinguishable from one another. Likewise, in every case, the similarity tests also suggest that niche dissimilarities are higher than expected by chance, which could lead to the rejection of the null hypothesis of niche conservatism (similarity) among lineages. There is no observed overlap of any *Eupherusa* species in E‐space, but the highest Schoener's *D* value is observed between species that are closer to each other in G‐ space, such as those within the SMS highlands (*E. cyanophrys* and *E. poliocerca*; Table 2).

### 3.2 Interspecific comparison among Sierra Madre del Sur lineages

**FIGURE 3:**
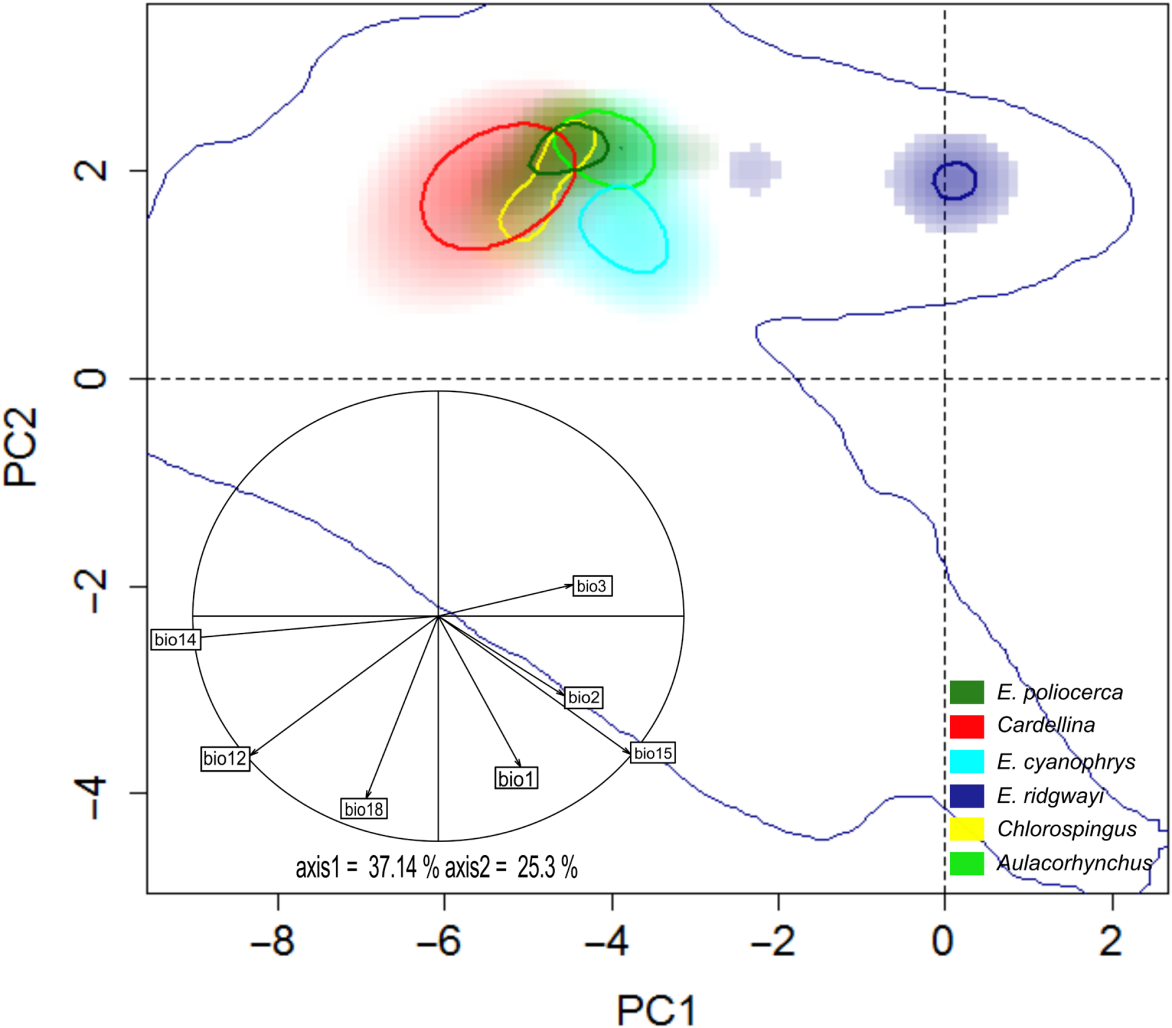
Overlap of the realized climatic niches of the different evaluated taxa from the Sierra Madre Sur. Color shading depicts the density of occurrences for each lineage by cell, color codes are as follows: *Aulacorhynchus* (green), *Chlorospingus* (yellow), *Cardellina* (red), *E. cyanophrys* (cyan), *E. poliocerca* (dark green) and *E. ridgwayi* (dark blue). The strong contours represent 10% of the highest values of occurrence density, whereas the thin lines represent 100% of the background available in the region. The correlation circle at the lower left of the figure shows the contribution of the climatic variables on the two axes of the PCA.

The first two PCA axes explained 62.44% of the original environmental variation (37.14% in PC1, and 25.3% in PC2; Figure 3). Overall, we observed marked differences in niche breadth in E‐space, in which the *Cardellina* SMS lineage occupies a wider climatic niche along PC1 and PC2 and even engulfs *Chlorospingus* and *E. poliocerca* occurrence densities. Conversely, *E. poliocerca* or *E. ridgwayi* exhibited a much narrower climatic niche, with *E. ridgwayi* being the most separated lineage in E‐space (Figure 3). We observed moderate overlap values between *Aulacorhynchus* versus *Chlorospingus* (*D* = 0.56) and *Aulacorhynchus* versus *E. cyanophrys* (*D* = 0.53). Contrarily, *E. ridgwayi* showed the lowest niche overlap values for all comparisons (Appendix S1, Table S1), while the *E. cyanophrys* versus *E. poliocerca* comparison showed a clear separation of both density clusters in E‐space (Figure 3) for niche overlap test (*D* = 0.0593; Appendix S1, Table S1, Figure S10).

The ordination null tests of niche equivalency showed that niche overlapping values were not significantly higher (*p >* .05) than the simulated values in 100% of the paired comparison cases. Therefore, the hypothesis that Grinnellian niches are identical can be rejected. Likewise, none of the background similarity tests were significantly higher than the simulated null distribution values (Appendix S1, Figure S11). Thus, both hypothesis of niche similarity (conservatism) among SMS lineages can be rejected.

### 3.3 Historical geographical distribution and areas of long‐term climatic stability

**FIGURE 4:**
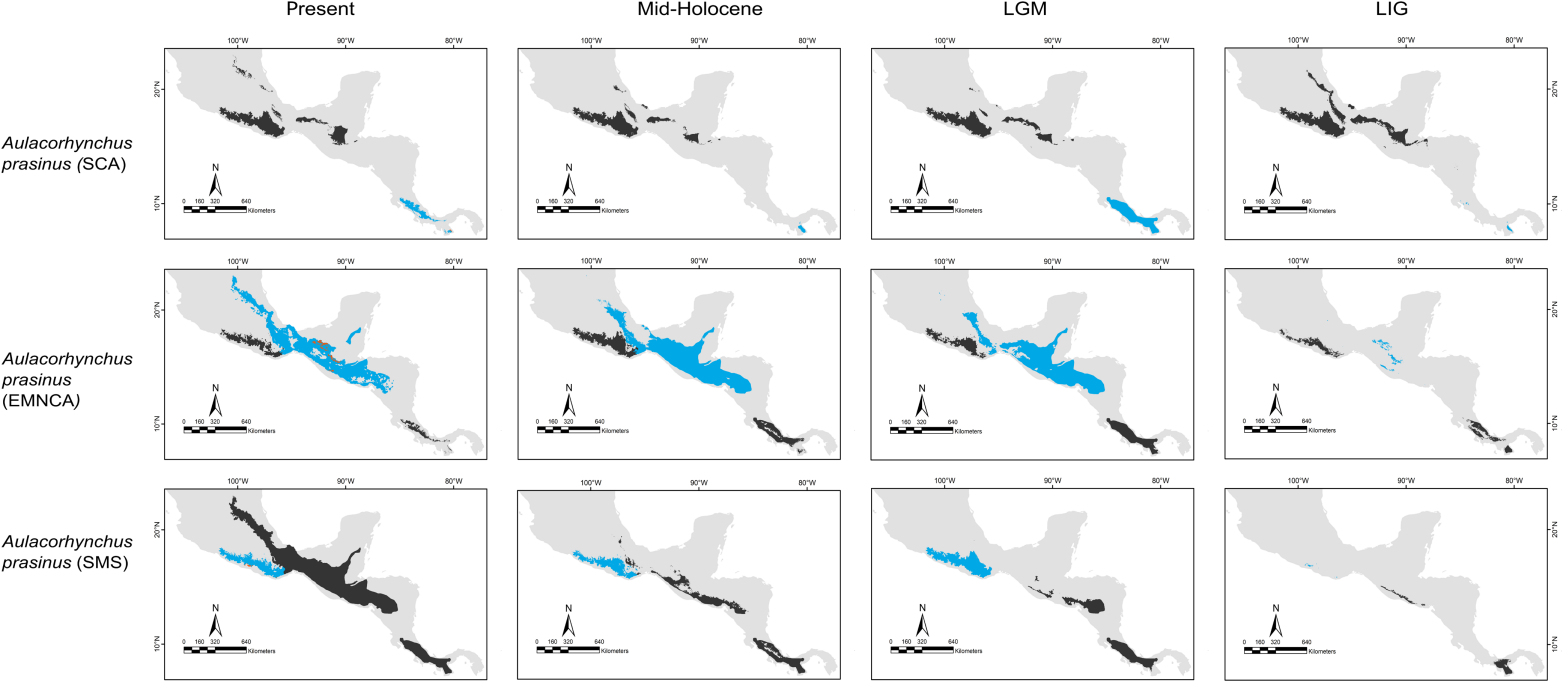
Binary projections of the potential geographic distributions of *Aulacorhynchus prasinus*, lineages estimated at the different Quaternary scenarios in the neotropics (blue), the assessed geographical matching for different lineages in each scenario (alloprediction, in dark gray) and the regions of historical climatic stability (orange).

**FIGURE 5:**
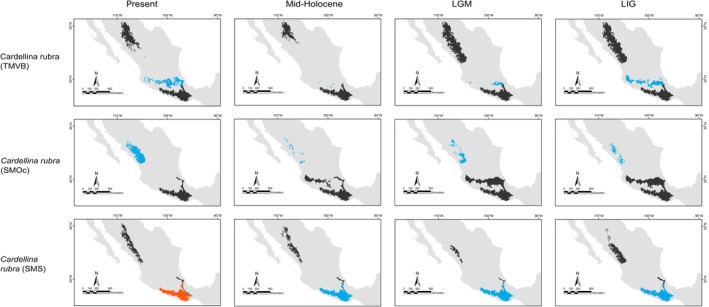
Binary projections of the potential geographic distributions of *Cardellina rubra* lineages estimated at the different Quaternary scenarios in the neotropics (blue), the assessed geographical matching for different lineages in each scenario (alloprediction, in dark gray), and the regions of historical climatic stability (orange).

**FIGURE 6:**
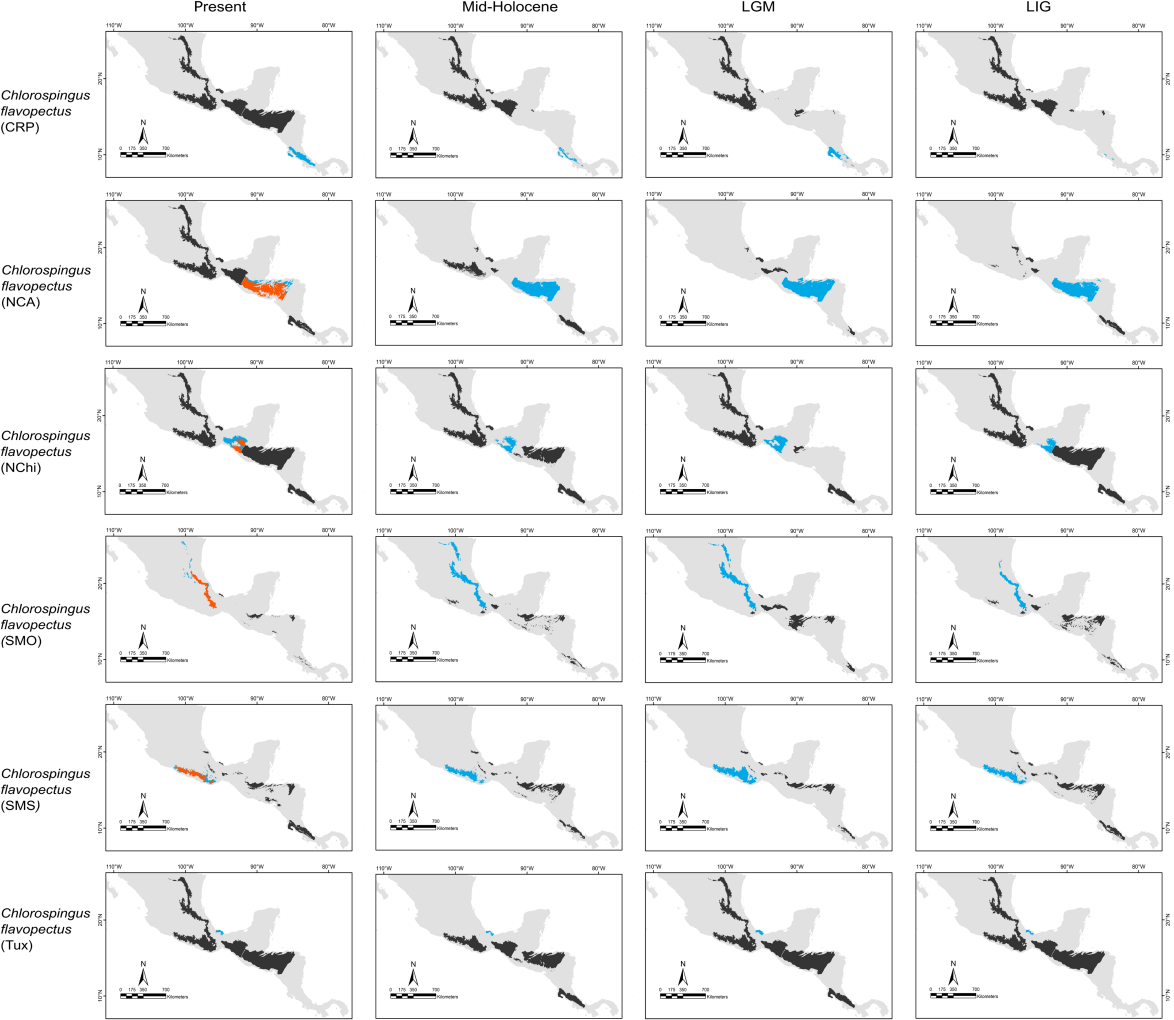
Binary projections of the potential geographic distributions of *Chlorospingus* lineages estimated at the different Quaternary scenarios in the neotropics (blue), the assessed geographical matching for different lineages in each scenario (alloprediction, in dark gray), and the regions of historical climatic stability (orange).

**FIGURE 7:**
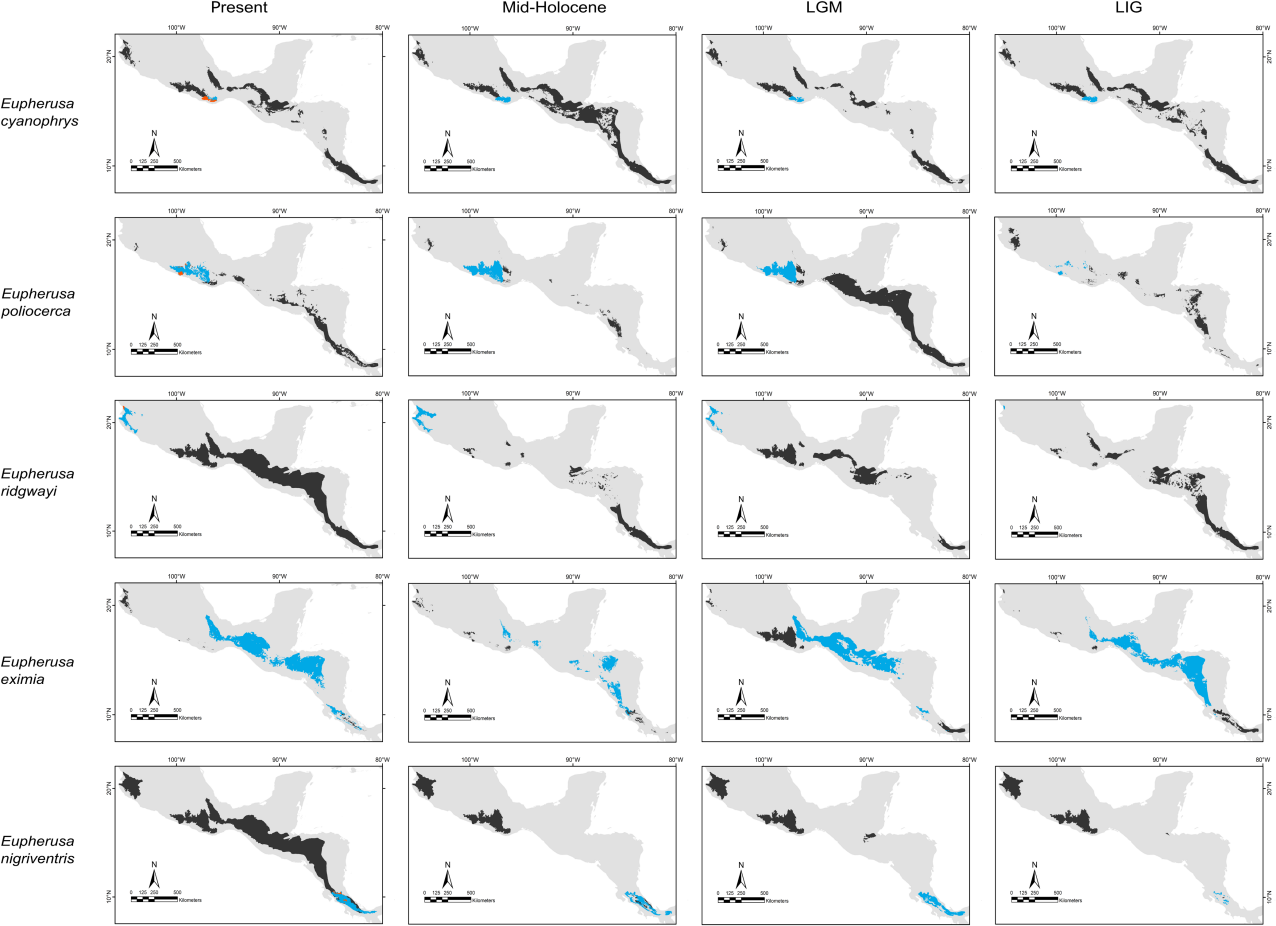
Binary projections of the potential geographic distributions of *Eupherusa* species estimated at the different Quaternary scenarios in the neotropics (blue), the assessed geographical matching for different lineages in each scenario (alloprediction, in dark gray), and the regions of historical climatic stability (orange).

Predicted distributions for each taxon based on analysis of the environmental data associated with occurrence data are shown in Figures 4, 5, 6, 7. All our niche models with ≥15 occurrence records performed better than a random estimate and were statistically descriptive of the climatic conditions influencing the geographic distribution of the analyzed lineages, with overall AUC values >0.7; whereas the lower observed p‐ROC value was 1.006 (*p* < .05; Table 1). Considering lineages with <15 occurrence records, jackknife evaluation tests showed high success rates (*q* > 0.7); nevertheless, only two (*E. poliocerca*, *E. cyanophrys*) of the five lineages evaluated were significant (*p* < .05; Appendix S2, Table S18).

**TABLE 1:** Statistics of model performance for the final models. Lineages with less than 15 occurrence records lack statistical values.

Taxon	Partial ROC	Omission rate (%)	AICc
*Aulacorhynchus*			
EMNCA (eastern Mexico‐north Central America)	1.104	6.34	3120.63
SCA (south Central America)	1.356	0.00	419.23
SMS (Sierra Madre del Sur)	—	—	—
*Chlorospingus*			
CRP (Costa Rica‐Panama)	1.192	27.27	447.16
NCA (north Central America)	1.079	29.41	802.66
NChi (northern Chiapas)	1.109	3.12	1374.9
SMO (Sierra Madre Oriental)	1.377	3.84	1138.78
SMS (Sierra Madre del Sur)	1.429	0.00	377.97
Tux (Tuxtlas Massif)	—	—	—
*Cardellina*			
SMOc (Sierra Madre Occidental)	1.474	22.20	407.56
SMS (Sierra Madre del Sur)	1.006	0.00	499.92
TMVB (Trans‐Mexican Volcanic Belt)	1.121	2.94	1534.15
*Eupherusa cyanophrys*	—	—	—
*E. poliocerca*	—	—	—
*E. eximia*	1.155	7.69	1282.94
*E. nigriventris*	1.160	8.33	1005.21
*E. ridgwayi*	—	—	—

Our predictions for current distributions in the analyzed lineages showed high correspondence with distinct mountain regions across Mesoamerica, however, geographical patterns in the paleodistribution models were not correspondent across all taxa. Predicted distributions during warmer epochs, such as the mid‐Holocene and the last interglacial, were similar across most taxa, showing fragmented geographic distributions in comparison to present or LGM scenarios. Overall, we observed that the LGM models showed less fragmented distribution areas and encompassed slightly larger geographic regions compared to the present (*Aulacorhynchus* and *Chlorospingus*; Figures 4 and 6). Contrarily, these models also revealed that lineages in *Cardellina* or *Eupherusa* (Figures 5 and 7) showed more fragmented geographic distributions in comparison to present day models.

Predicted areas of long‐term climatic stability across Mesoamerica, within the current known distribution of our selected lineages, were almost continuously distributed along Northern Central America to the SMS and eastern regions of Mexico, with predicted larger sizes for the *Chlorospingus* lineages in comparison to the other analyzed taxa. We also observed a pair of noticeable exceptions for *C. flavopectus* (NCA lineage) and *C. rubra* (SMS lineage) for which apparently no major distributional changes occurred throughout time; along with an important geographical retraction of conditions defining the niche of *C. rubra* (SMOc & TMVB lineages) or *E. eximia*, which were localized into allopatric areas mainly during the Mid‐Holocene (Figures 4, 5, 6, 7). Although models for each taxon predicted different locations of overall stability through time, three main areas were consistently recovered among the studied taxa that span regions where phylogeographic breaks are located: within the SMS (mainly in western Oaxaca), the southeastern region of Mexico, and the mountains across Central America. We also found an overall pattern of lineages with large autoprediction such as *Chlorospingus* in the SMO or SMS regions, suggesting null or minimal overlap between them. Likewise, lineages such as *Chlorospingus* (Tux) and *Eupherusa* (*E. cyanophrys*), which have an overall maximum area occupancy of 2000 km^2^ in the present, predicted large suitable conditions towards other nearby regions occupied by phylogenetically close lineages.

## 4 DISCUSSION

The uplift of several mountain ranges in Mesoamerica undoubtedly had consequences for mechanisms affecting diversification events, which include geographic isolation of populations, and the formation and change of climatic and precipitation regimes that may have promoted ecological isolation (Mastretta‐Yanes et al., 2015, 2018). This latter has been evidenced by research focused on patterns of genetic differentiation of montane Neotropical birds (e.g., Arbeláez‐Cortés et al., 2014; Carnaval et al., 2009; Klicka et al., 2011; Licona‐Vera et al., 2018), however, these events may predate the genetic divergence of lineages within the analyzed taxa (Rocha‐Méndez et al., 2019). Smith et al. (2014) proposed a model of avian diversification in which speciation is strongly related to lineages persistence and move through a landscape previously structured by large scale tectonic/geologic processes. This is consistent with the idea that lineages with low vagility are expected to accrue differentiation at higher rates than more dispersive lineages (Wakeley & Aliacar, 2001). Correlations between lineage ages or Quaternary climatic events reported elsewhere (Castillo‐Chora, Sánchez‐González, et al., 2021; Castillo‐Chora, Zamudio‐Beltrán, et al., 2021; Espinosa‐Chávez et al., 2024), suggest that landscape and environmental change are an important component in diversification; nevertheless, the idiosyncratic life histories and lineage‐specific attributes are the primary determinants of species diversity (Burney & Brumfield, 2009), which may lead to incongruent distribution patterns.

We have identified environmental constraints for the distribution of four Mesoamerican bird taxa through inferring niche divergence and overlap (Broennimann et al., 2012; Di Cola et al., 2017), which represent a significant advance and further support for previously reported patterns of genetic divergence among our selected allopatric lineages (e.g., Barrera‐Guzmán et al., 2012; Bonaccorso et al., 2008, 2011; Hernández‐Baños et al., 2012; Puebla‐Olivares et al., 2008; Sánchez‐González et al., 2007). Differences in the environmental constraints across altitudinal and latitudinal gradients would be indicative of niche evolution, as distinctive ecophysiological tolerances and trait changes in morphology or behavior begin to accumulate before considerable changes in niches are observed (Silva et al., 2020).

Our findings on climatic niche overlap suggest that lineages have acquired their own ecological identity throughout Mesoamerica by undergoing differentiation from shared ancestral habitat preferences. This is supported by lineages distributed in the same geographic region across different latitudes (such as *E. ridgwayi, E. poliocerca* and *E. cyanophrys* in the SMS, Figure 2d), or those which are isolated but still in geographical proximity to each other (such as *Chlorospingus* in Los Tuxtlas and SMO, Figure 2b). Clinal niche differences for the SMS *Eupherusa* lineages partially mirror a regionalization of subprovinces for the SMS (Morrone, 2017), within which a large variability in vegetation type and structure, topography, wind speed, radiation or cloud cover may influence the local atmospheric moisture which in turn may influence the distribution of organisms across the region.

Our niche overlap results suggest some variability in the environmental space inhabited by different Mesoamerican avian taxa (Figure 2). Closely related lineages in *Chlorospingus* and *Cardellina* from the SMS and NChi or TMVB respectively (Barrera‐Guzmán et al., 2012; Bonaccorso et al., 2008, 2011; Bonaccorso & Guayasamin, 2013; García‐Moreno et al., 2004; Hernández‐Baños et al., 2012; Maldonado‐Sánchez et al., 2016; Puebla‐Olivares et al., 2008; Rocha‐Méndez et al., 2019; Sánchez‐González et al., 2007; Weir et al., 2008; Winker, 2016) differed in their occupied niche space despite non‐significant niche overlap *D* values (Table 2). This supports that niches tend to remain constant through the phylogeny in the short or medium terms, and thus ecological divergence could be the result of complex geographical and ecological events (Eaton et al., 2008; McCormack et al., 2010; Mota‐Vargas & Rojas‐Soto, 2016; Warren et al., 2008). Likewise, some E‐space niche comparisons indicated that lineages such as *Aulacorhynchus* from “EMNCA” (Figure 2a) or *Chlorospingus* from “NChi” (Figure 2b), occupy a broader range of climatic conditions than other lineages. Within *Chlorospingus*, we observe a contrasting pattern where niches are partially overlapping or completely nested within others (Figure 2b). Such a pattern of overlap and partial nesting is mainly recovered when comparing different taxa distributed in the same ecoregion at very similar latitudes (Figure 3).

**TABLE 2:** Summary of the niche equivalence/similarity analyses using a principal component analysis (PCA‐env) and Schoener's (1970) overlap.

Niche overlap	Empirical Schoener's *D* value	Niche equivalence results (*p*‐values)	Niche similarity results (*p‐*values) A–B/B–A
*Aulacorhynchus prasinus*			
EMNCA‐SCA	0.142	.52	.70/0.58
EMNCA‐SMS	0.053	.90	.82/0.94
SCA‐SMS	0.000	1	1/1
*Chlorospingus flavopectus*			
CRP‐NCA	0.133	.009*	.68/0.53
CRP‐SMO	0.054	.009*	.74/0.67
CRP‐Tux	0.173	.06	.80/0.86
CRP‐NChi	0.035	.37	.55/0.62
CRP‐SMS	0.000	1	1/1
NCA‐SMO	0.061	.38	.50/0.34
NCA‐Tux	0.159	.70	.82/0.70
NCA‐Nchi	0.042	.81	.933/0.64
NCA‐SMS	0.088	.82	.94/1
SMO‐Tux	0.025	1	1/1
SMO‐Nchi	0.071	1	1/1
SMO‐SMS	0.000	1	1/1
Tux‐Nchi	0.007	1	1/1
Tux‐SMS	0.000	1	1/1
Nchi‐SMS	0.068	.95	.93/1
*Cardellina rubra*			
TMVB‐SMOc	0.199	.13	.13/0.20
TMVB‐SMS	0.154	.87	.82/0.71
SMOc‐SMS	0.033	.96	.94/1
*Eupherusa* spp.			
*E. cyanophrys–E. poliocerca*	0.110	.76	.84/0.87
*E. cyanophrys–E. eximia*	0.007	.74	1/1
*E. cyanophrys–E. nigriventris*	0.003	1	1/1
*E. cyanophrys–E. ridgwayi*	0.056	.94	1/1
*E. poliocerca–E. eximia*	0.000	1	1/1
*E. poliocerca–E. nigriventris*	0.000	1	1/1
*E. poliocerca–E. ridgwayi*	0.029	1	1/1
*E. eximia–E. nigriventris*	0.081	.88	.85/0.81
*E. eximia–E. ridgwayi*	0.000	1	1/1
*E. nigriventris–E. ridgwayi*	0.000	1	1/1

*Note*: EMNCA (eastern Mexico‐north Central America), SCA (south Central America), SMS (Sierra Madre del Sur), CRP (Costa Rica‐Panama), NCA (north Central America), NChi (northern Chiapas), Tux (Los Tuxtlas Massif), SMO (Sierra Madre Oriental), SMOc (Sierra Madre Occidental), TMVB (Trans‐Mexican Volcanic Belt). Histograms for niche comparison analyses are presented in Appendix S1 (Figures S4 and S5). An * depicts *p* < 0.05.

Despite the archipelago‐like distribution of the humid montane forests within the SMS region offers different conditions (e.g. regional differences in latitudinal and altitudinal vegetation composition), which have been described as one of the main triggers of diversification and in situ speciation (Challenger & Soberón, 2008; Morrone, 2017), we observed moderate niche overlap among taxa (Figure 3). Such an overlap may be explained by the fact that *Aulacorhynchus*, *Chlorospingus* and *Cardellina* SMS lineages occupy broader and overall similar areas, whereas *E. poliocerca* and *E. cyanophrys* occupy only isolated patches throughout the region. The small distribution area of *E. cyanophrys* may be the reason for the non‐overlapping pattern with the niche space of other lineages, thus suggesting specialization to the overall similar conditions across the region. Moreover, the isolated *E. ridgwayi* showed null overlap or similarity with the other SMS taxa, suggesting that *E. ridgwayi* may be adapted to different environmental niche conditions. The E‐space niche comparisons mirror the degree to which species may share or compete for resources in each area, supporting that a lower niche overlap may be expected because of mutual exclusion (Pastore et al., 2021; Tsafack et al., 2021).

Projections represent a spatially explicit estimation of habitat suitability. Our predictions for current distributions in the analyzed lineages showed high correspondence with distinct mountain regions across Mesoamerica, however, geographical patterns in the paleodistribution models were not correspondent across all taxa. Our niche models also agreed with previous phylogeographic studies of the target taxa in which habitat isolation and connectivity cycles might have a differential impact on the ecological drift (i. e., random fluctuation of species abundances [Fodelianakis et al., 2021; Gilbert & Levine, 2017]) of birds depending on the elevational range. For example, *Eupherusa* species tend to range to the lower limits of humid montane forests, whereas the range of *Cardellina* is reported to change from higher humid pine‐oak forests during the breeding season to lower oak forests in winter, and thus may be considered an altitudinal migrant (Billerman et al., 2022; Howell & Webb, 1995). Our paleo‐distribution models suggest that taxa with different maximum altitudes presented different range stability, fragmentation, and size between Quaternary periods.

During the Quaternary, climate is known to have undergone notable changes that had a decisive influence on the genetic structure of populations in several organisms, due to alternated periods of isolation and connectivity (Masta, 2000; Mastretta‐Yanes et al., 2015; Silva et al., 2014). Our findings lack a clear pattern for the evolutionary impacts of Pleistocene range shifts in Mesoamerica, especially when single species are considered, suggesting that each taxon responded differently to forest shifts, following their climatic requirements and tolerances. According to the moist forest model (Ramírez‐Barahona & Eguiarte, 2013), the humidity conditions favored a down‐slope migration of montane forest species and population connectivity between mountain ranges during cold glacial periods. However, our observations of some lineages showing smaller projected ranges during the LGM was unexpected and disagree with this model and the previous evidence showing that montane organisms had larger ranges during this period (e.g., Antonelli et al., 2018; Ornelas et al., 2010, 2013). Despite this, our study is not the first to show forest species with fragmented ranges during the LGM (see Bonaccorso et al., 2006). It is also interesting that several climatic stability areas were very small (<50 km in linear distance), which may have caused the species disjunct geographic ranges, supporting allopatry as a factor causing differentiation between the analyzed lineages (McCormack et al., 2010).

Overall, our results show wide predictions in northern Chiapas ‐ northern Central America; these predictions and observed overlap in geographic and environmental space, as well as their allopredictive power, may be the result of the predominant types of vegetation (e.g. *Quercus, Pinus*) from acid soils of volcanic origin (Challenger & Soberón, 2008) such as those in the Central American Mountain systems and its northern projection represented by the Chiapas Sierra Madre, the Chimalapas, and the Central Chiapas massif (Morrone, 2019; Whattam & Stern, 2016). Our results also suggest that lineages such as *Chlorospingus* from Los Tuxtlas or *E. ridgwayi* from the westernmost SMS, despite being isolated, predict similar potential distribution ranges to the other lineages showing ecological congruency between them, these past and present requirements for the lineages establishment being highly similar suggest niche conservatism (Peterson, 2001). As these taxa are geographically restricted, they experience a limited range of environmental variation; nevertheless, they have high allopredictive power which resembles a “nested niche” pattern (i. e., populations with specialized niche requirements that are nested within the requirements of other populations; Peterson & Holt, 2003). Thus, a more refined characterization of the niches of the lineages analyzed herein would require detailed and spatially extensive field studies. Further work should doubtlessly include variables beyond the climatic spectrum that we have used herein (i.e., vegetation cover, altitude, measures of food availability, or species interactions; Moreno‐Contreras et al., 2020).

The dynamic climatic conditions associated with altitudinal and latitudinal variation in disjunct and phenotypically differentiated lineages between mountain ranges in Mesoamerica observed in our results (e.g., Avendaño et al., 2013; Navarro‐Sigüenza et al., 2001; Sánchez‐González et al., 2007) may be as important as the physical barriers limiting gene flow between populations, given that forests from the eastern and western slopes possess contrasting conditions (Alcántara et al., 2002; García‐Franco et al., 2008; Lauer, 1973; Luna‐Vega et al., 2001; Velázquez et al., 2000). Our documentation of ecological differentiation, combined with previous evidence for morphological and genetic differentiation supports the idea that lineages in the analyzed taxa are independently evolving. As pointed out by Leaché et al. (2009), populations may diverge genetically, morphologically, or ecologically, thus providing the operational criteria useful for species delimitation. If distinct criteria are coincident for differentiation, then there is stronger support for lineage distinctiveness, thus highlighting the importance of the sky‐island dynamics during the Pleistocene, with climatic factors such as temperature and precipitation as key elements determining lineage boundaries.

## AUTHOR CONTRIBUTIONS

**Alberto Rocha‐Méndez:** Conceptualization (lead); data curation (lead); formal analysis (lead); investigation (lead); writing – original draft (equal); writing – review and editing (equal). **David A. Prieto‐Torres:** Conceptualization (supporting); formal analysis (equal); funding acquisition (supporting); methodology (equal); writing – original draft (equal); writing – review and editing (equal). **Luis A. Sánchez‐González:** Conceptualization (supporting); formal analysis (equal); methodology (supporting); writing – original draft (equal); writing – review and editing (equal). **Adolfo G. Navarro‐Sigüenza:** Conceptualization (lead); formal analysis (lead); investigation (lead); resources (lead); writing – original draft (equal); writing – review and editing (equal).

## CONFLICT OF INTEREST STATEMENT

The authors declare no conflict of interest.

## Supporting information


Appendix S1.

Appendix S2.

## Data Availability

The data that support the findings of this study are available in Global Biodiversity Information Facility (http://www.gbif.org). URLs of downloaded data are depicted in Appendix S1, Table S2. Files containing the filtered and analyzed data, along with the associated R codes to make the E‐env analyses are deposited on Github (github.com/rochamendez/ClimaticNicheShifts_MesoamericanBirds).
