# Supplementary material for: Climatic niche shifts and ecological sky‐island dynamics in Mesoamerican montane birds
Source: Ecol Evol. 2024 Sep 4;14(9):e70236. doi: 10.1002/ece3.70236 (PMC11374531; doi:10.1002/ece3.70236)
Supplement: Supplementary file 1 — Appendix S1. [file ECE3-14-e70236-s002.docx]

**SUPPLEMENTARY MATERIAL I**

Figure S1. Correlation plots for the taxa used in this study: *Aulacorhynchus, Chlorospingus, Cardellina*, and *Eupherusa* (*E. cyanophrys, E. eximia, E. poliocerca, E. nigriventris*, and *E. ridgwayi*). The variables resume the environmental information for the given threshold (r <0.8). Each taxon has a plot which is divided into two insets. Top insets depict correlation plots, the highly correlated variables are shown in blue. Bottom insets depict a dendogram cluster analysis; at least one bioclimatic variable was selected for each branch arranged in each cluster. Variables selected for subsequent analyses in *Aulacorhynchus* were: bio1(annual mean temperature), bio2 (mean diurnal range), bio3 (isothermality), bio12 (annual precipitation), bio14 (precipitation of the driest month), bio18 (precipitation of warmest quarter) and bio19 (precipitation of the coldest quarter). Selected variables for *Chlorospingus* were: bio1, bio2, bio3, bio12, bio15 (precipitation seasonality), bio18, bio19. Variables for *Cardellina* were: bio1, bio2, bio3, bio12, bio14, bio15, bio18, bio19. Variables for *E. cyanophrys* were: bio1, bio2, bio3, bio8 (mean temperatura of wettest quarter), bio10 (mean temperatura of warmest quarter), bio12, bio13 (precipitation of wettest month), bio14, bio15. Variables for *E. eximia* were: bio1, bio2, bio3, bio12, bio15, bio18, bio19. Variables for *E. poliocerca* were: bio1, bio2, bio8, bio12, bio18, bio19. Variables for *E. nigriventris* were: bio1, bio2, bio3, bio4 (temperatura seasonality), bio5 (max temperatura of warmest month), bio10, bio11 (mean temperatura of coldest quarter), bio12, bio13, bio15, bio18. Variables for *E. ridgwayi* were: bio1, bio2, bio3, bio5, bio12, bio14, bio19.

Figure S2. Correlation plots for the five *Eupherusa* analyzed species presence points in the present study. Variables that resume environmental information for the given threshold (r < 0.8). A) Correlation plot depicting variables that are highly correlated in blue. B) Dendrogram cluster analysis, at least one bioclimatic variable was selected for each branch arranged in each cluster. Variables selected for subsequent analyses were bio 1 (annual mean temperature), bio 2 (mean diurnal range), bio 3 (isothermality), bio 12 (annual precipitation), bio 15 (precipitation seasonality), and bio 18 (precipitation of warmest quarter).

Figure S3. Correlation plots for SMS lineages presence points, lineages correspond to *Aulacorhynchus, Chlorospingus, Cardellina, E. cyanophrys, E. poliocerca*, and *E. ridgwayi* taxa. Variables that resume environmental information for the given threshold (r < 0.8). A) Correlation plot depicting variables that are highly correlated in blue. B) Dendrogram cluster analysis, at least one bioclimatic variable was selected for each branch arranged in each cluster. Variables selected for subsequent analyses were bio 1 (annual mean temperature), bio 2 (mean diurnal range), bio 3 (isothermality), bio 4 (temperature seasonality), bio 12 (annual precipitation), bio 14 (precipitation of the driest month), bio 15 (precipitation seasonality), and bio 18 (precipitation of warmest quarter).

Figure S4. Observed niche overlap between analyzed lineages within A) *A. prasinus* and B) *C. flavopectus*, bars with a diamond (red) and simulated niche overlaps (grey bars) on which tests of niche equivalency are calculated from 100 iterations.

Figure S5. Observed niche overlap between analyzed lineages within A) *C. rubra* and B) *Epherusa*, bars with a diamond (red) and simulated niche overlaps (grey bars) on which tests of niche equivalency are calculated from 100 iterations.

Fig. S6. Results obtained from the environmental niche analyses, depicting the climatic conditions occupied by *Aulacorhynchus prasinus* in all its known distributional ranges. Shading shows the density of the occurrences by cell. The solid and dashed contour lines illustrate 100 and 50% of the available environment. Colors indicate genetic clusters evaluated: eastern Mexico and north Central America (EMNCA) represented in dark blue, south-Central America (SCA) in cyan, and Sierra Madre del Sur (SMS) in red.

Fig. S7. Results obtained from the environmental niche analyses, depicting the climatic conditions occupied by *Chlorospingus flavopectus* in all its known distributional ranges. Shading shows the density of the occurrences by cell. The solid and dashed contour lines illustrate 100 and 50% of the available environment. Colors indicate genetic clusters evaluated: Costa Rica-Panama (CRP) represented in cyan, northern Central America (NCA) in orange, northern Chiapas (NChi) in yellow, Sierra Madre Oriental (SMO) in blue, Sierra Madre del Sur (SMS) in red and Tuxtlas Massif (Tux) in green.

Fig. S8. Results obtained from the environmental niche analyses, depicting the climatic conditions occupied by *Cardellina rubra* in all its known distributional ranges. Shading shows the density of the occurrences by cell. The solid and dashed contour lines illustrate 100 and 50% of the available environment. Colors indicate genetic clusters evaluated: Sierra Madre Occidental (SMOc) in dark blue, Sierra Madre del Sur (SMS) in red, and the Trans Mexican Volcanic Belt (TMVB) in brown.

Fig. S9. Results obtained from the environmental niche analyses, depicting the climatic conditions occupied by *Eupherusa* genus in its distributional range, colors indicate distinct species. Shading shows the density of the occurrences by cell. The solid and dashed contour lines illustrate 100 and 50% of the available environment for the analyzed species.

Figure S10. Results obtained from the environmental niche analyses, depicting the climatic conditions occupied by SMS lineages. Shading shows the density of the occurrences by cell. The solid and dashed contour lines illustrate 100 and 50% of the available environment. Colors indicate genetic lineages of the different evaluated taxa which inhabit the SMS ecoregion: *Aulacorhynchus* represented in green, *Chlorospingus* in yellow, *Cardellina* in red, *E. cyanophrys* in cyan, *E. poliocerca* depicted in dark green and *E. ridgwayi* in dark blue.

Figure S11. Observed niche overlap D values between analyzed taxa inhabiting the SMS region, bars with a diamond (red) and simulated niche overlaps (grey bars) on which tests of niche equivalency are calculated from 100 iterations.

Table S1. Niche overlap D and Similarity I values of lineages inhabiting the SMS province derived from niche equivalency tests calculated through 100 iterations.

Table S2. Access numbers and date of download for GBIF records of the taxa analyzed in the present study. The clean data bases can be found in: github.com/rochamendez/ ClimaticNicheShifts_MesoamericanBirds.


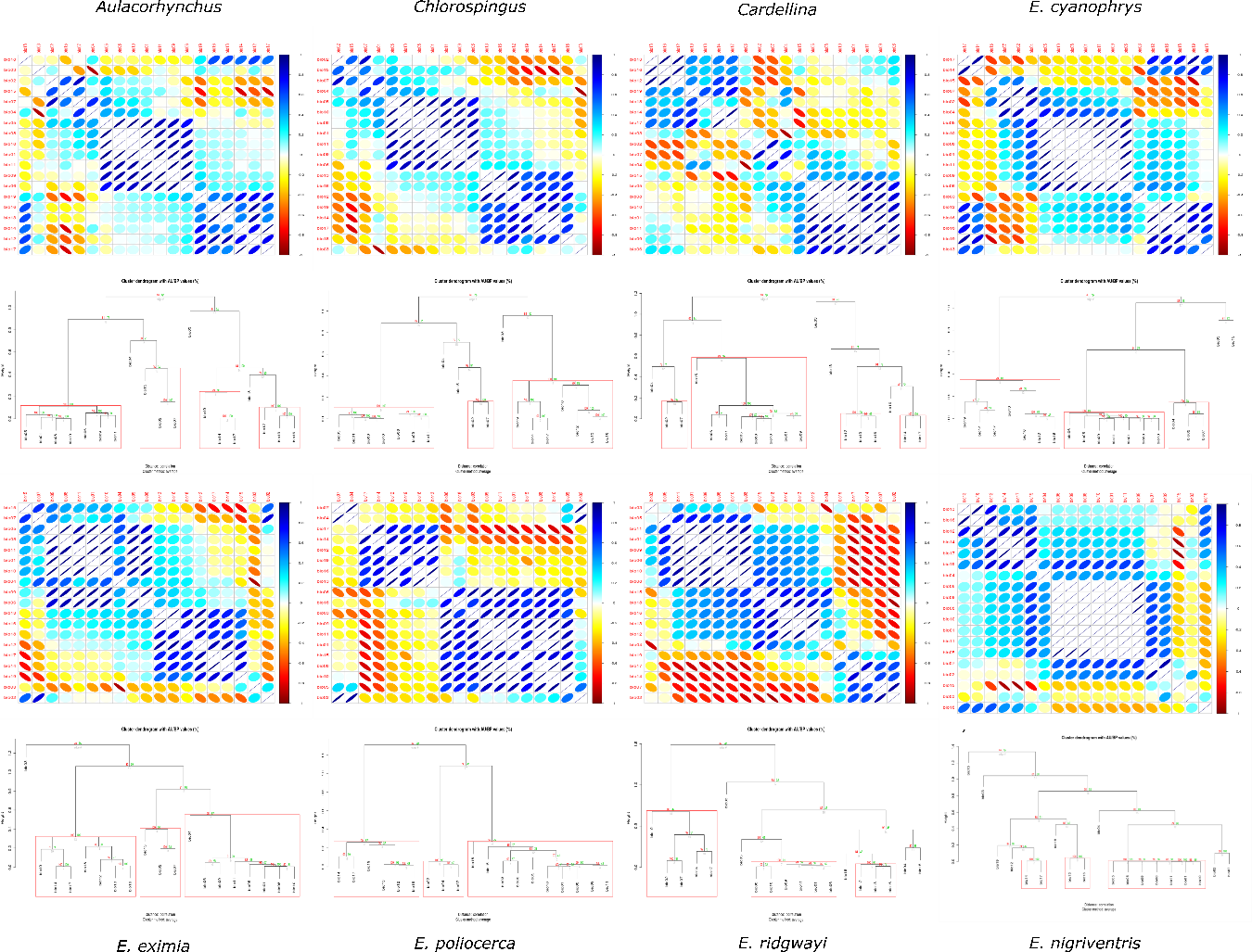


Fig. S1


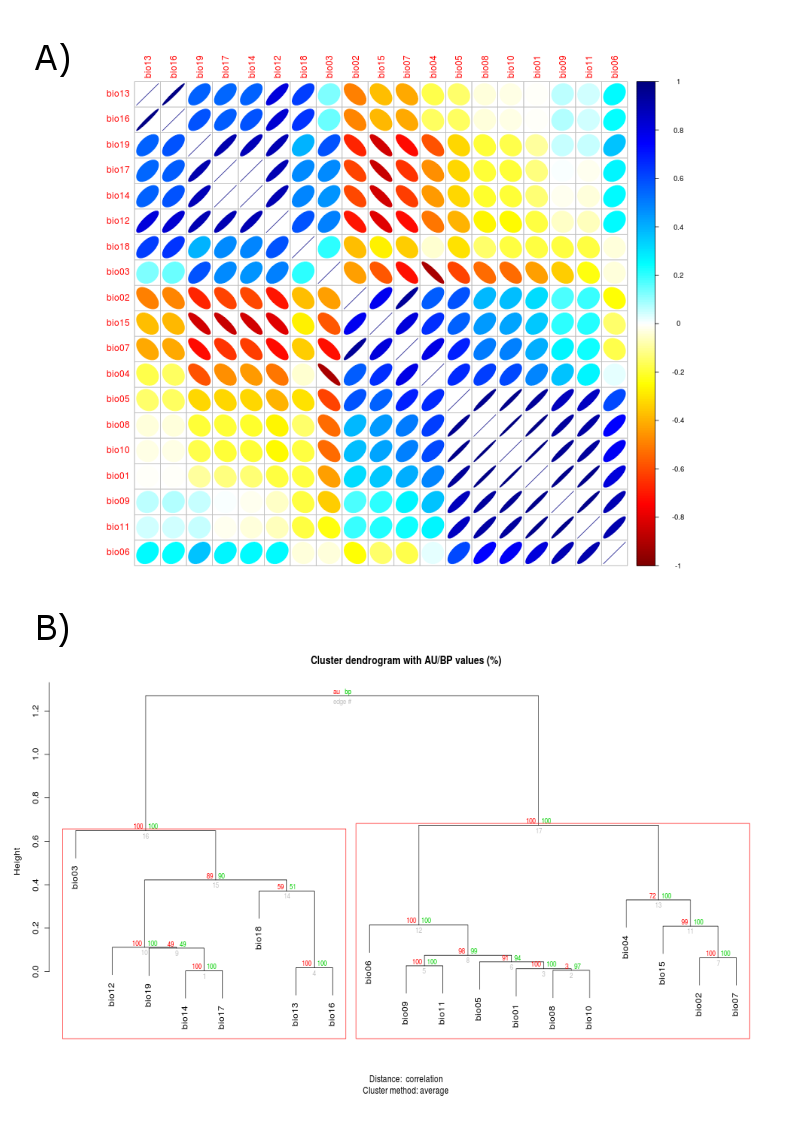


Fig. S2.


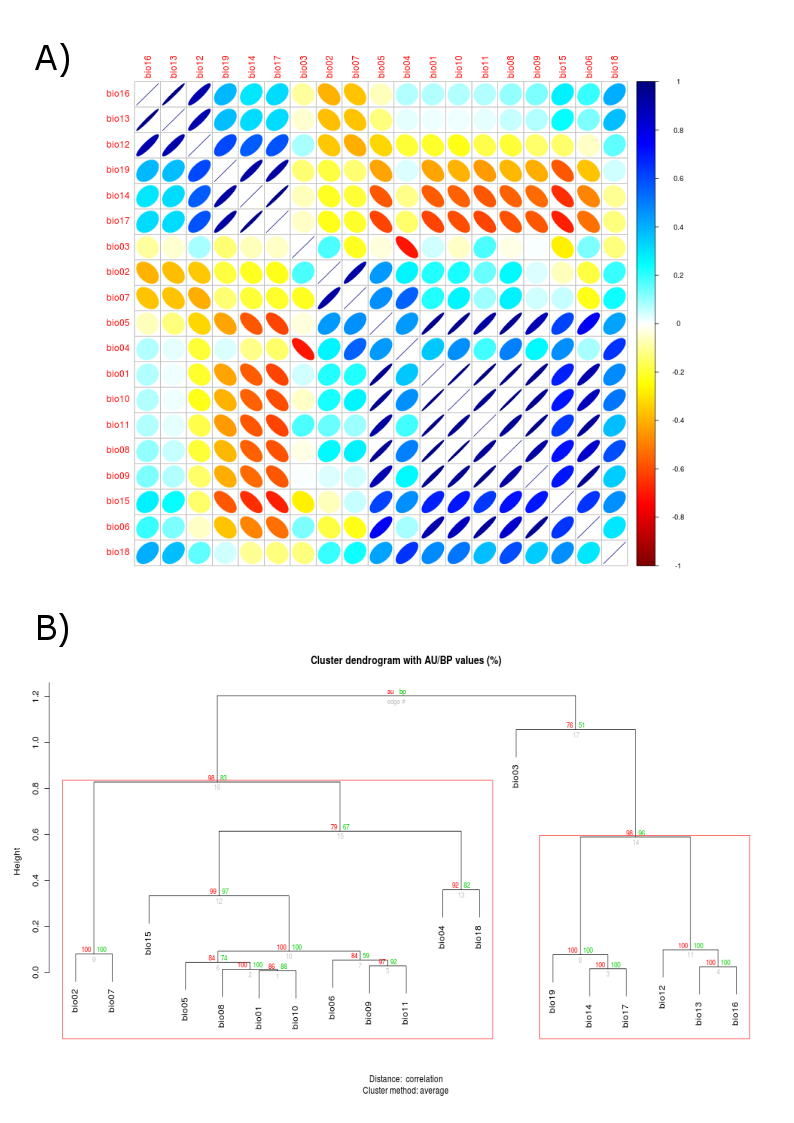


Fig. S3.


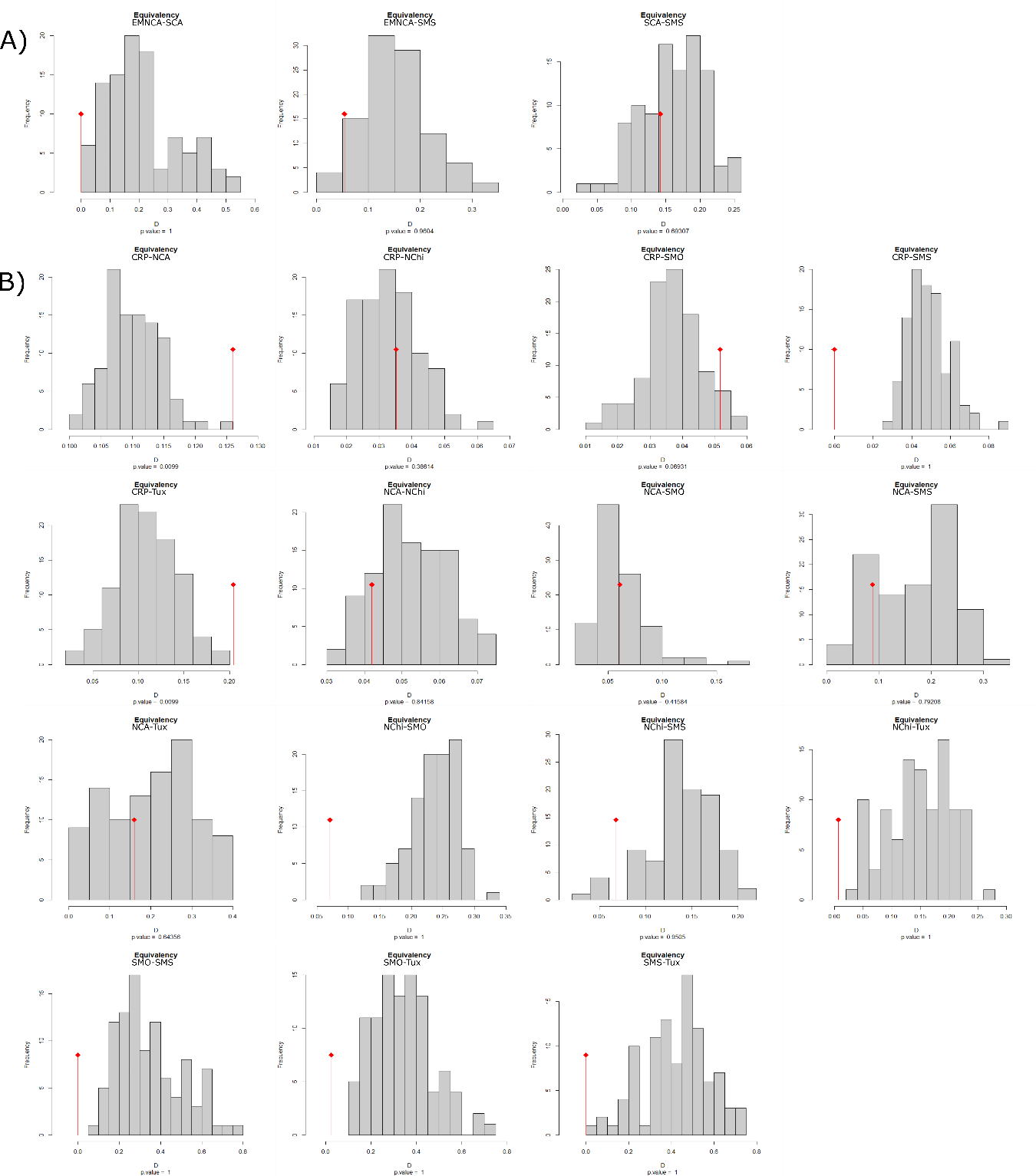


Fig. S4.


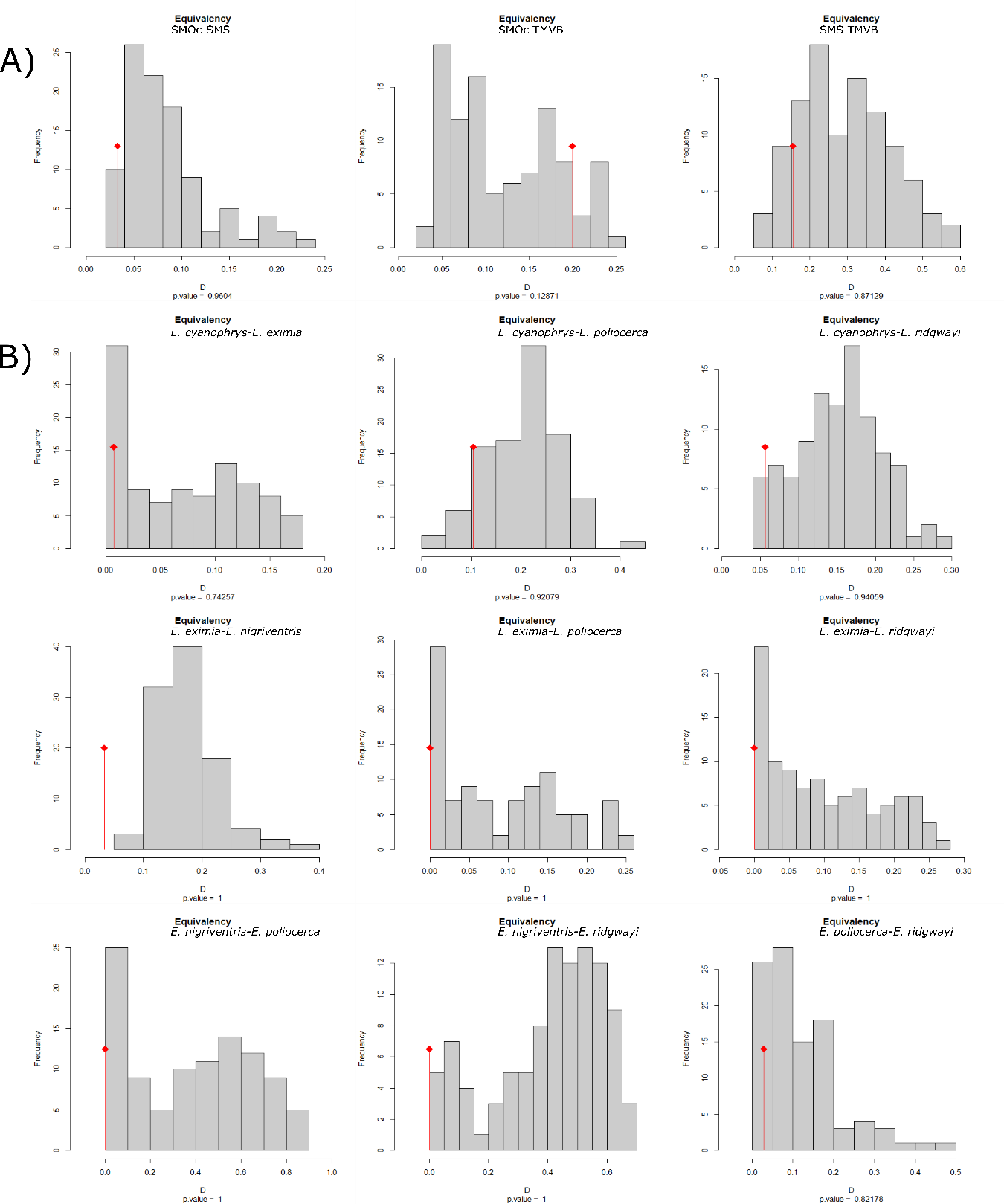


Fig. S5.


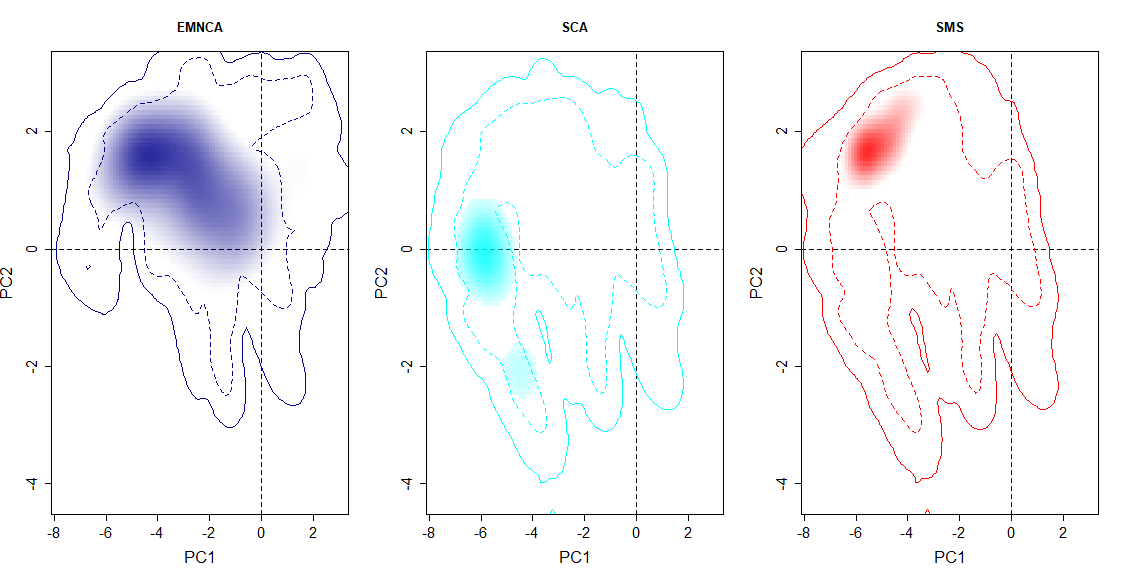


Fig. S6.


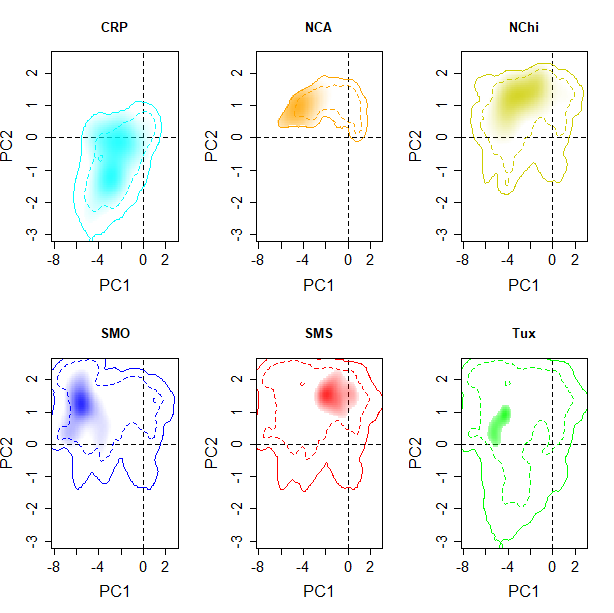


Fig. S7.


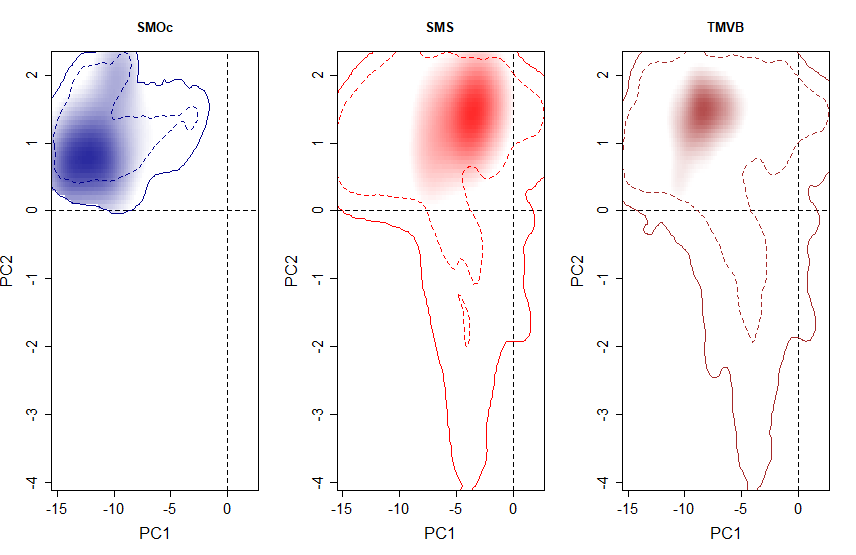


Fig. S8.


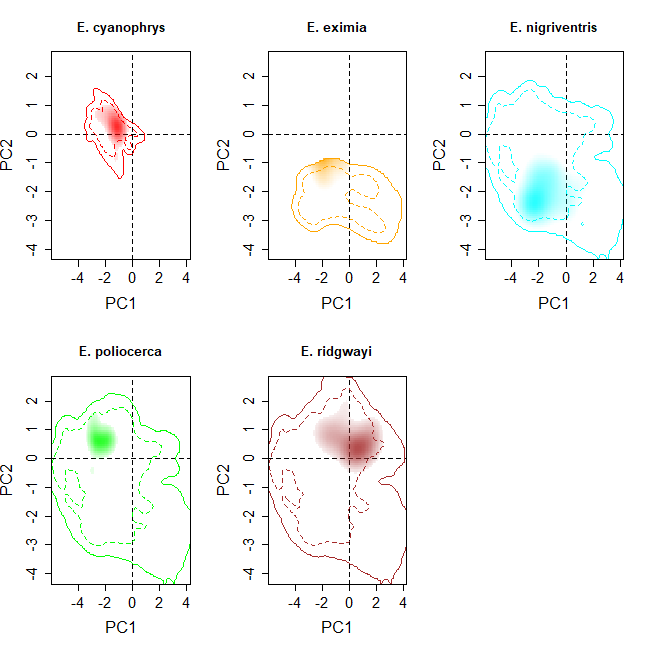


Fig. S9.


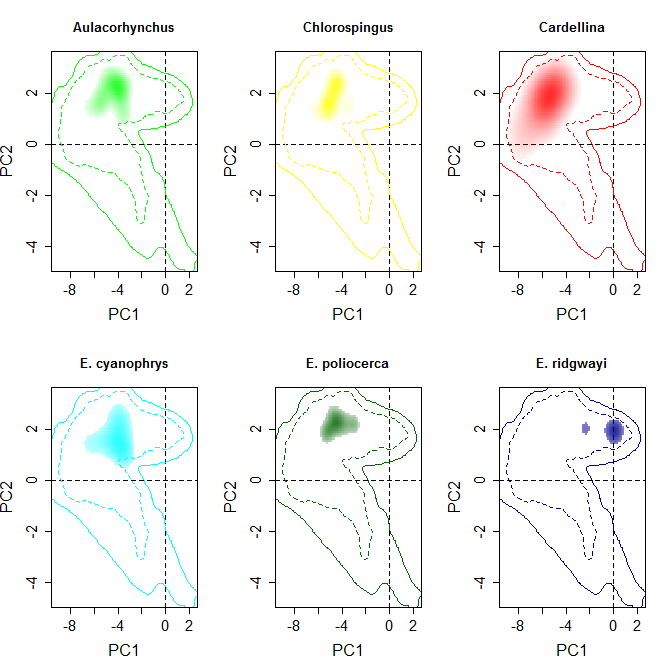


Fig. S10.


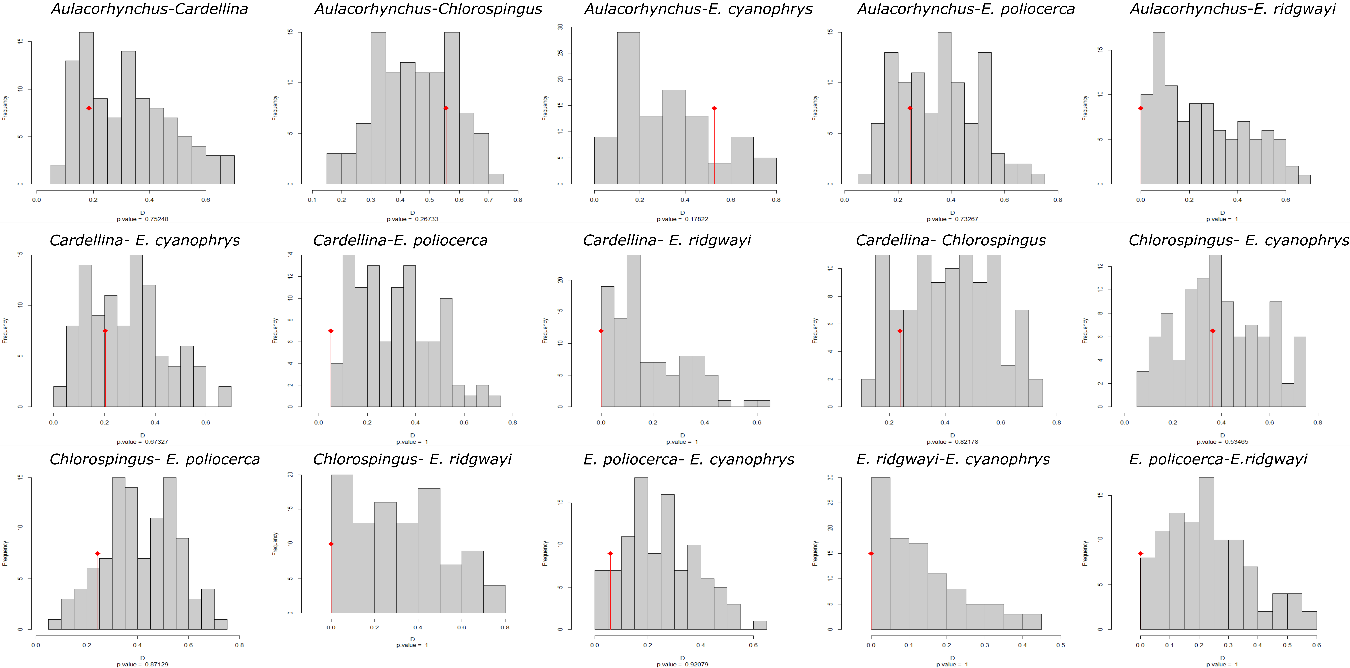


Fig. S11.

| SMS taxa | **Empirical Schoener’s *D* value** | **Niche equivalence results (P-values)** | **Niche similarity results (P-values)**  **A-B / B-A** |
| --- | --- | --- | --- |
| *Aulacorhynchus* vs *Chlorospingus* | 0.5549 | 0.26733 | 0.30693/0.28793 |
| *Aulacorhynchus* vs *Cardellina* | 0.1852 | 0.75248 | 0.86139/0.82915 |
| *Aulacorhynchus* vs *E*. *cyanophrys* | 0.5284 | 0.17822 | 0.16832/0.1872 |
| *Aulacorhynchus* vs *E. poliocerca* | 0.2461 | 0.73267 | 0.53465/0.643 |
| *Aulacorhynchus* vs *E. ridgwayi* | 0 | 1 | 1/1 |
| *Chlorospingus* vs *Cardellina* | 0.2384 | 0.82178 | 0.81188/0.8 |
| *Chlorospingus* vs *E. cyanophrys* | 0.3647 | 0.53465 | 0.47525/0.5 |
| *Chlorospingus* vs *E. poliocerca* | 0.2430 | 0.87129 | 0.85149/0.87964 |
| *Chlorospingus* vs *E. ridgwayi* | 0 | 1 | 1/1 |
| *Cardellina* vs *E. cyanophrys* | 0.2031 | 0.67327 | 0.83168/0.784 |
| *Cardellina* vs *E. poliocerca* | 0.0508 | 1 | 1/1 |
| *Cardellina* *vs E. ridgwayi* | 0 | 1 | 1/1 |
| *E. cyanophrys* vs *E. poliocerca* | 0.0593 | 0.92079 | 0.92079/0.92079 |
| *E. cyanophrys* vs *E. ridgwayi* | 1.1102e^-16^ | 1 | 1/1 |
| *E. poliocerca* vs *E. ridgwayi* | 0.00221 | 1 | 1/1 |

Table S1

| **Taxon** | **GBIF Occurrence Download** | **Date** |
| --- | --- | --- |
| ***Aulacorhynchus*** | https://doi.org/10.15468/dl.cmvsmj | 07.09.2018 |
| ***Chlorospingus*** | https://doi.org/10.15468/dl.ijd4af | 07.09.2018 |
| ***Cardellina*** | https://doi.org/10.15468/dl.xoostd | 07.09.2018 |
| ***Eupherusa cyanophrys*** | https://doi.org/10.15468/dl.52daoj | 07.09.2018 |
| ***E. poliocerca*** | <https://doi.org/10.15468/dl.gu1nfh> | 07.09.2018 |
| ***E. eximia*** | https://doi.org/10.15468/dl.7nfmhg | 07.09.2018 |
| ***E. nigriventris*** | https://doi.org/10.15468/dl.pyogc9 | 04.13.2019 |
| ***E. ridgwayi*** | https://doi.org/10.15468/dl.t19hei | 07.12.2018 |

Table S2
