# Supplementary material for: Climatic niche shifts and ecological sky‐island dynamics in Mesoamerican montane birds
Source: Ecol Evol. 2024 Sep 4;14(9):e70236. doi: 10.1002/ece3.70236 (PMC11374531; doi:10.1002/ece3.70236)
Supplement: Supplementary file 2 — Appendix S2. [file ECE3-14-e70236-s001.docx]

**SUPPLEMENTARY MATERIAL II**

Figure S1. Calibration results for the distinct taxa analyzed, and the best candidate models for A) *A. prasinus* (EMNCA), B) *A. prasinus* (SCA), C) *C. rubra* (SMOc), D) *C. rubra* (SMS), E) *C. rubra* (TMVB), F) *E. eximia*, G) *E. nigriventris*, H) *C. flavopectus* (CRP).

Figure S2. Calibration results for the distinct taxa analyzed, and the best candidate models for A) *C. flavopectus* (NCA), B) *C. flavopectus* (NChi), C) *C. flavopectus* (SMO), D) *C. flavopectus* (SMS).

Table S1. Relative contributions of the environmental variables to the Maxent model of *A. prasinus* (EMNCA).

Table S2. Relative contributions of the environmental variables to the Maxent model of *A. prasinus* (SCA)*.*

Table S3. Relative contributions of the environmental variables to the default Maxent model of *A. prasinus* (SMS)*.*

Table S4. Relative contributions of the environmental variables to the Maxent model of *C. rubra* (SMOc).

Table S5. Relative contributions of the environmental variables to the Maxent model of *C. rubra* (SMS).

Table S6. Relative contributions of the environmental variables to the Maxent model of *C. rubra* (TMVB).

Table S7. Relative contributions of the environmental variables to the Maxent model of *C. flavopectus* (CRP)*.*

Table S8. Relative contributions of the environmental variables to the Maxent model of *C. flavopectus* (NCA).

Table S9. Relative contributions of the environmental variables to the Maxent model of *C. flavopectus* (NChi)*.*

Table S10. Relative contributions of the environmental variables to the Maxent model of *C. flavopectus* (SMO)*.*

Table S11. Relative contributions of the environmental variables to the Maxent model of *C. flavopectus* (SMS)*.*

Table S12. Relative contributions of the environmental variables to the default Maxent model of *C. flavopectus* (Tux)*.*

Table S13. Relative contributions of the environmental variables to the default Maxent model of *E. cyanophrys.*

Table S14. Relative contributions of the environmental variables to the default Maxent model of *E. poliocerca.*

Table S15. Relative contributions of the environmental variables to the Maxent model of *E. ridgwayi.*

Table S16. Relative contributions of the environmental variables to the Maxent model of *E. eximia.*

Table S17. Relative contributions of the environmental variables to the Maxent model of *E. nigriventris.*

Table S18. Jackknife tests and corresponding p-values computed for assessing predictability of niche models in low-sample size lineages as described in Pearson et al. (2007).


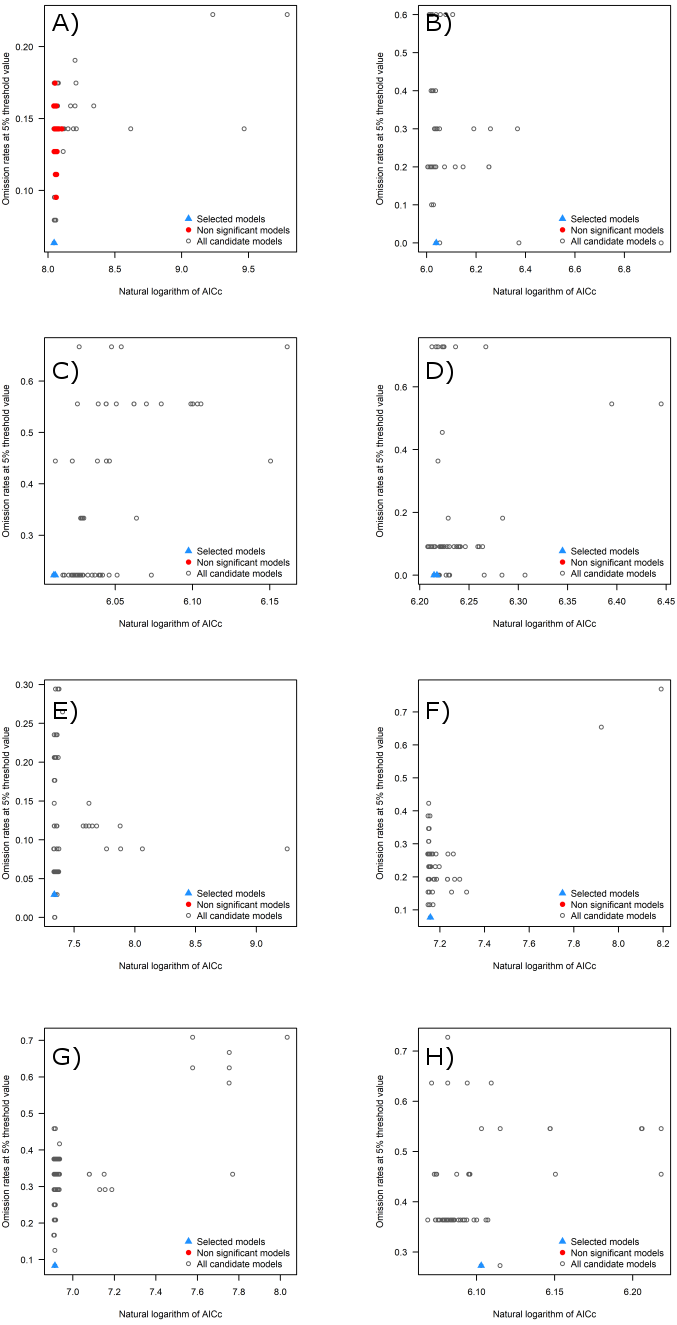


Fig. S1.


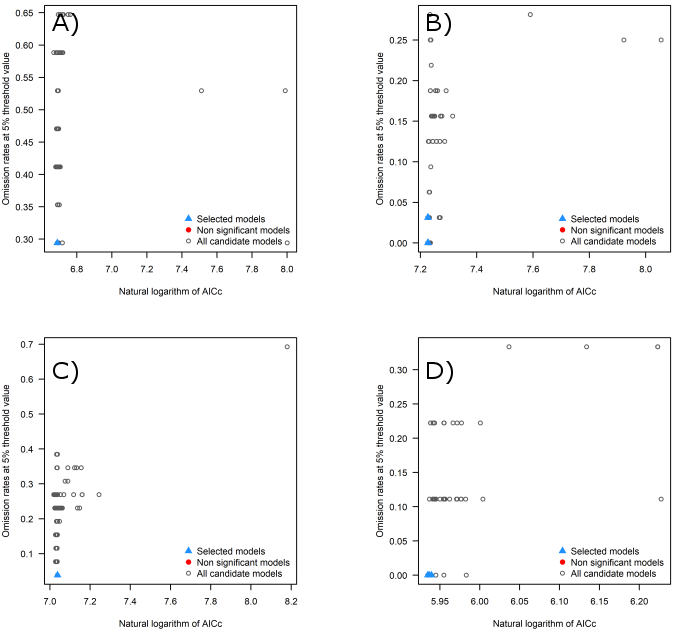


Fig. S2.

| Variable | Percent contribution |
| --- | --- |
| Bio1 | 21.2 |
| Bio12 | 18.3 |
| Bio3 | 16.2 |
| Bio4 | 13.8 |
| Bio2 | 12.1 |
| Bio15 | 10.3 |
| Bio14 | 8.2 |

Table S1.

| Variable | Percent contribution |
| --- | --- |
| Bio1 | 64.7 |
| Bio12 | 16.2 |
| Bio4 | 8.3 |
| Bio14 | 8.2 |
| Bio15 | 2 |
| Bio3 | 0.5 |
| Bio2 | 0.2 |

Table S2.

| Variable | Percent contribution |
| --- | --- |
| Bio3 | 52.4 |
| Bio2 | 33.9 |
| Bio12 | 13.5 |
| Bio4 | 0.1 |
| Bio14 | 0 |
| Bio15 | 0 |
| Bio1 | 0 |

Table S3.

| Variable | Percent contribution |
| --- | --- |
| Bio3 | 73.8 |
| Bio12 | 6.1 |
| Bio1 | 5.8 |
| Bio2 | 4.9 |
| Bio19 | 4.8 |
| Bio14 | 2.5 |
| Bio15 | 1.1 |
| Bio18 | 1.1 |

Table S4.

| Variable | Percent contribution |
| --- | --- |
| Bio2 | 46.8 |
| Bio14 | 20 |
| Bio19 | 19.7 |
| Bio1 | 12.9 |
| Bio3 | 0.7 |
| Bio15 | 0 |
| Bio12 | 0 |
| Bio18 | 0 |

Table S5.

| Variable | Percent contribution |
| --- | --- |
| Bio2 | 48.6 |
| Bio1 | 22.3 |
| Bio3 | 18.6 |
| Bio12 | 4.1 |
| Bio15 | 2.9 |
| Bio19 | 1.7 |
| Bio18 | 1.2 |
| Bio14 | 0.6 |

Table S6.

| Variable | Percent contribution |
| --- | --- |
| Bio1 | 45.4 |
| Bio18 | 13.1 |
| Bio12 | 12.2 |
| Bio3 | 8.7 |
| Bio15 | 7.8 |
| Bio2 | 6.7 |
| Bio19 | 6.1 |

Table S7.

| Variable | Percent contribution |
| --- | --- |
| Bio1 | 69.1 |
| Bio19 | 10.9 |
| Bio3 | 10 |
| Bio12 | 0 |
| Bio2 | 0 |
| Bio18 | 0 |
| Bio15 | 0 |

Table S8.

| Variable | Percent contribution |
| --- | --- |
| Bio1 | 40.2 |
| Bio19 | 33.8 |
| Bio3 | 20.5 |
| Bio2 | 4.1 |
| Bio18 | 1.4 |
| Bio15 | 0.1 |
| Bio12 | 0 |

Table S9.

| Variable | Percent contribution |
| --- | --- |
| Bio2 | 75.5 |
| Bio18 | 7.5 |
| Bio12 | 6.6 |
| Bio19 | 4.5 |
| Bio3 | 3.3 |
| Bio1 | 1.6 |
| Bio15 | 1 |

Table S10.

| Variable | Percent contribution |
| --- | --- |
| Bio2 | 38 |
| Bio12 | 25.5 |
| Bio18 | 10.6 |
| Bio19 | 5.2 |
| Bio15 | 0.6 |
| Bio1 | 0 |
| Bio3 | 0 |

Table S11.

| Variable | Percent contribution |
| --- | --- |
| Bio3 | 86.3 |
| Bio15 | 9.9 |
| Bio18 | 2.7 |
| Bio1 | 1.1 |
| Bio2 | 0 |
| Bio12 | 0 |
| Bio19 | 0 |

Table S12.

| Variable | Percent contribution |
| --- | --- |
| Bio12 | 69 |
| Bio3 | 9.6 |
| Bio15 | 5.1 |
| Bio2 | 2 |
| Bio18 | 0 |
| Bio1 | 0 |

Table S13.

| Variable | Percent contribution |
| --- | --- |
| Bio3 | 64.2 |
| Bio18 | 20.5 |
| Bio1 | 13.2 |
| Bio12 | 1.4 |
| Bio15 | 0.5 |
| Bio2 | 0.1 |

Table S14.

| Variable | Percent contribution |
| --- | --- |
| Bio18 | 34.3 |
| Bio15 | 32.5 |
| Bio1 | 26.9 |
| Bio2 | 5.2 |
| Bio3 | 0.6 |
| Bio12 | 0.4 |

Table S15.

| Variable | Percent contribution |
| --- | --- |
| Bio3 | 38.7 |
| Bio18 | 20.5 |
| Bio2 | 15 |
| Bio12 | 10.9 |
| Bio15 | 10.5 |
| Bio1 | 4.5 |

Table S16.

| Variable | Percent contribution |
| --- | --- |
| Bio12 | 27.9 |
| Bio1 | 19.3 |
| Bio2 | 18.7 |
| Bio18 | 14.4 |
| Bio3 | 14.3 |
| Bio15 | 5.5 |

Table S17.

| **Lineage** | **Success rate (q)** | **p-value** |
| --- | --- | --- |
| Aulacorhynchus (SMS) | 0.9 | 0.3078 |
| Chlorospingus (Tux) | 0.9 | 0.5993 |
| E. cyanophrys | 0.714286 | 0.5593 |
| E. poliocerca | 0.8888 | 0.002 |
| E. ridgwayi | 0.75 | 0.0045 |

Table S18.
